# Developmental Stability: A Major Role for *Cyclin G* in *Drosophila melanogaster*

**DOI:** 10.1371/journal.pgen.1002314

**Published:** 2011-10-06

**Authors:** Vincent Debat, Sébastien Bloyer, Floria Faradji, Nelly Gidaszewski, Nicolas Navarro, Pablo Orozco-terWengel, Valérie Ribeiro, Christian Schlötterer, Jean S. Deutsch, Frédérique Peronnet

**Affiliations:** 1 Muséum National d'Histoire Naturelle Département Systématique et Evolution UMR 7205, Centre National de la Recherche Scientifique, Paris, France; 2 Laboratoire de Biologie du Développement UMR 7622, Université Pierre et Marie Curie-Paris 6, Centre National de la Recherche Scientifique, Paris, France; 3 Laboratory of Artificial and Natural Evolution Department of Zoology and Animal Biology, University of Geneva Sciences III, Geneva, Switzerland; 4 Institut für Populationsgenetik, Veterinärmedizinische Universität Wien, Vienna, Austria; Georgia Institute of Technology, United States of America

## Abstract

Morphological consistency in metazoans is remarkable given the pervasive occurrence of genetic variation, environmental effects, and developmental noise. Developmental stability, the ability to reduce developmental noise, is a fundamental property of multicellular organisms, yet its genetic bases remains elusive. Imperfect bilateral symmetry, or fluctuating asymmetry, is commonly used to estimate developmental stability. We observed that *Drosophila melanogaster* overexpressing *Cyclin G* (*CycG*) exhibit wing asymmetry clearly detectable by sight. Quantification of wing size and shape using geometric morphometrics reveals that this asymmetry is a genuine—but extreme—fluctuating asymmetry. Overexpression of *CycG* indeed leads to a 40-fold increase of wing fluctuating asymmetry, which is an unprecedented effect, for any organ and in any animal model, either in wild populations or mutants. This asymmetry effect is not restricted to wings, since femur length is affected as well. Inactivating *CycG* by RNAi also induces fluctuating asymmetry but to a lesser extent. Investigating the cellular bases of the phenotypic effects of *CycG* deregulation, we found that misregulation of cell size is predominant in asymmetric flies. In particular, the tight negative correlation between cell size and cell number observed in wild-type flies is impaired when *CycG* is upregulated. Our results highlight the role of *CycG* in the control of developmental stability in *D. melanogaster*. Furthermore, they show that wing developmental stability is normally ensured via compensatory processes between cell growth and cell proliferation. We discuss the possible role of *CycG* as a hub in a genetic network that controls developmental stability.

## Author Summary

Developing organisms face genetic, environmental, and stochastic variations, and yet their ability to reach stereotyped phenotypes is pervasive. Although genetic bases of this robustness are actively investigated, no consensus has been achieved, some authors attributing it to particular genes such as *Hsp90*, while others to emerging properties of complex genetic networks. A particularly puzzling component of robustness is developmental stability, i.e. the reduction of developmental noise. Genetic bases of developmental stability have remained equally elusive. In bilaterians, developmental stability is commonly measured by fluctuating asymmetry (FA), where the same organ is compared on both sides of the same individual. Here, we show that ubiquitous overexpression of the *Cyclin G* gene induces a 40-fold increase of wing size FA, which is an unprecedented effect, for any organ and in any animal model. Investigating the cellular bases of this asymmetry, we show that the tight negative correlation between cell size and cell number that occurs in wild-type flies was lost. Since not only wings but also legs were affected, *Cyclin G* appears a candidate gene for a general mechanism of developmental stability in Drosophila, suggesting that phenotypic robustness can be strongly influenced by individual genes.

## Introduction

Precision of developmental processes is of great evolutionary importance since it conditions the accurate replication of the selected phenotype. Stabilizing selection is thus thought to favor robust developmental systems [1]. Waddington was the first to suggest that the ability to buffer variation – referred to as developmental homeostasis – is a fundamental property of organisms [2]. He divided developmental homeostasis into two subcomponents: canalization that buffers genetic and environmental effects, and developmental stability that buffers developmental noise [3]. Despite numerous speculations about the evolutionary role of such buffering processes, their study has remained marginal to the mainstream of evolutionary biology until precise molecular processes were identified that might account for these properties. On the one hand, studies on the role of *Hsp90* [4], [5] and more recently of other *Hsp* genes [6] and microRNAs [7], [8], in the buffering of genetic variation, contributed to the idea that robustness is ensured by specific genes and genetic processes. On the other hand, complex genetic networks can be intrinsically robust to perturbations [9], questioning the existence of specific robustness genes. To accommodate these contradictory results, it was suggested that some genes of particular importance for robustness might exist as hubs in complex networks [10]. Whether these results apply to developmental stability is not known [11]–[13].

Developmental stability is commonly estimated by fluctuating asymmetry (FA). The two sides of bilaterally symmetrical traits are influenced by the same genes and environmental conditions and thus only differ by developmental noise. Genetics of developmental stability and FA has been controversial [14]. The vast majority of studies report that the additive genetic variation for FA is extremely low or non significant. These low values have been interpreted either as a low signal to noise ratio, FA values being typically very small, or suggesting a non-additive genetic basis [14]. In line with the latter hypothesis, a few QTL interacting epistatically for FA have been detected in mouse (reviewed in [14]). The best documented case of an individual gene affecting FA is the homologue of the *Drosophila Notch* gene in the sheep blowfly (*Lucilia cuprina*) [15]. Its effects on FA are however limited to bristles [16], a trait known to be controlled by *Notch*. Hence, no evidence for the existence of genes controlling developmental stability in a general way has been reported so far.

**Figure 1:**
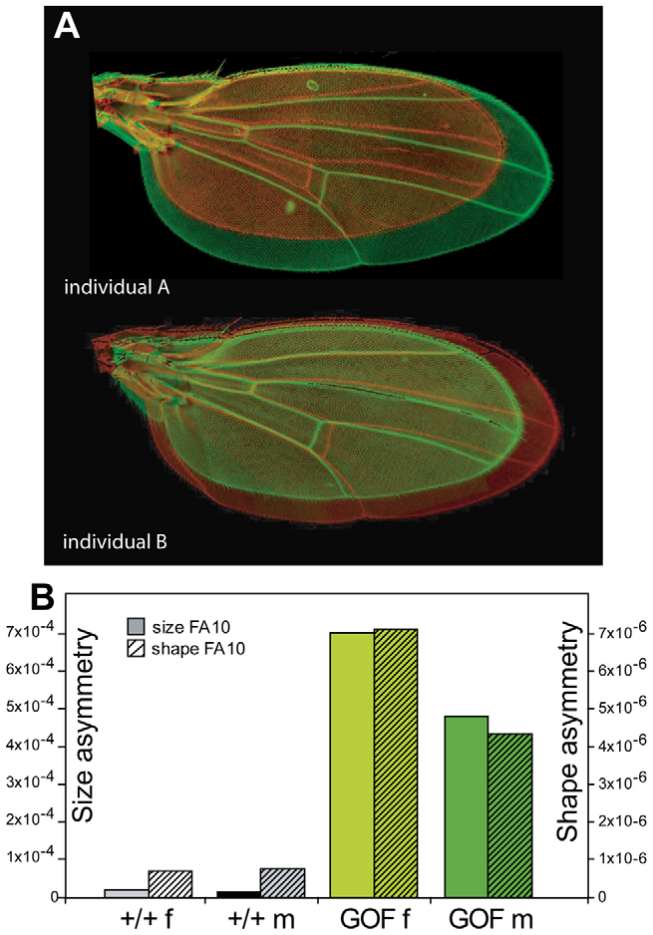
Extreme wing fluctuating asymmetry in flies overexpressing *CycG*. A: Two asymmetrical individuals overexpressing *CycG* ubiquitously in a *w^1118^* background under the control of the *daughterless* (*da*) driver. Photos of the two wings are superimposed: in red the left wing and in green the right wing. B: Effect of *CycG* overexpression on wing size FA (open bars) and shape FA (dashed bars). The progeny of crosses between *da::Gal4/+* females and *RCG76/+* males in *w^1118^* background was analyzed (see Tables S2 and S3 for values). Only *+/+* and GOF (*da::Gal4>RCG76*) individuals are represented here. FA10: FA index corrected for measurement error and directional asymmetry (see Material and Methods) [26]. Grey: +/+ females; black: +*/+* males; light green: GOF females; dark green: GOF males.

Cyclins are a family of proteins primarily characterized as cell cycle regulators (for review see [17]). Nevertheless, of the 14 cyclins currently characterized, only a few (Cyclins A, B, D, and E) appear to be major actors of the cell cycle [18]. Other cyclins, such as Cyclins C, K, H or T, are involved in transcriptional processes (for review see [19]). We have recently shown that *Drosophila* Cyclin G is an unconventional cyclin involved, on the one hand, in transcriptional regulation *via* the Trithorax and Polycomb co-factor Corto [20], [21] and, on the other hand, in regulation of cell growth and cell cycle [22]. *CycG* is thus suspected to play a major role in *Drosophila* development. Ubiquitous overexpression of the *Drosophila Cyclin G* (*CycG*) gene during development induces high lethality. Escaper flies exhibit various mild phenotypes: they are smaller, have a longer developmental time, reduced eyes and rotated genitalia [22]. Most remarkably, although wings are normally structured, they exhibit a striking pattern of size asymmetry that fluctuates in the population (Figure 1A). This last effect suggested that *CycG* might be a good candidate gene for the control of developmental stability in *Drosophila*. Below, we detail the effects of overexpressing or RNAi-inactivating *CycG* on FA, integrating the analyses at the macroscopic and cellular levels.

## Results

The UAS/Gal4 system was used to overexpress or RNAi-inactivate *CycG* during development [21], [22]. In order to minimize genetic and environmental sources of phenotypic variation, we used nearly isogenic backgrounds and conducted the experiments in a carefully standardized environment (see Material and Methods). The progeny was analyzed for wing size and shape using geometric morphometrics [23], [24]. Sample sizes are shown in Table 1.

**Table 1:** Sample size.

Genetic background	Driver	Experiment	Genotype	females	males	Total
*w^1118^*	*da*	*GOF*	*da/+*	48	46	94
			*RCG76/+*	48	48	96
			*da>RCG76*	53	55	108
			*+/+*	47	47	94
*yw^67c23^*	*da*	*GOF*	*da/+*	49	48	97
			*RCG76/+*	48	50	98
			*da>RCG76*	25	26	51
			*+/+*	50	47	97
	*da*	*LOF*	*da>dsCycG2*	45	44	89
			*+/+*	48	50	98
	*Act*	*GOF*	*Act>RCG76*	34	40	74
			*+/+*	49	50	99
	*sd*	*GOF*	*sd>RCG76*	45	50	95
			*+/+*	49	50	99
Total				638	651	1289

### Overexpressing *CycG* alters wing size, wing shape, and increases fluctuating asymmetry

**Figure 2:**
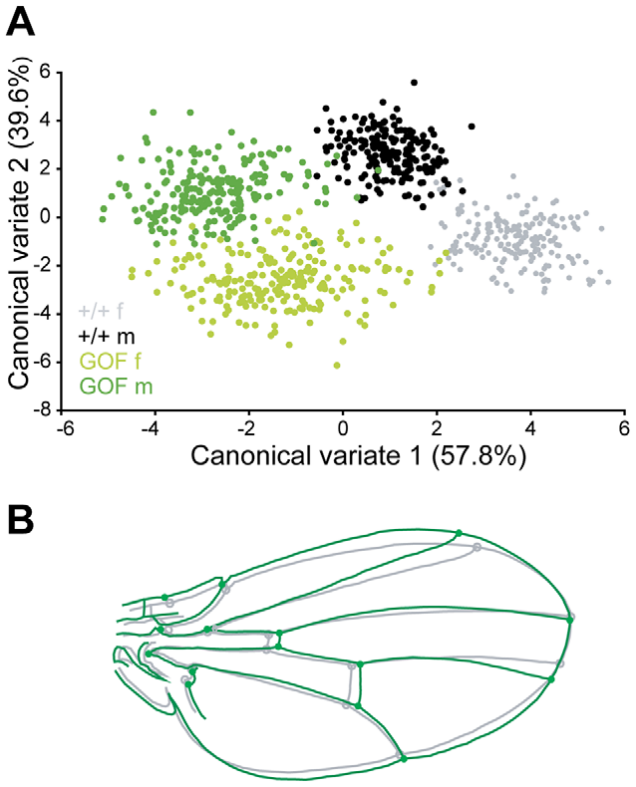
Overexpressing *CycG* alters wing shape. A: Discriminant analysis of wing shape data. The same individuals as in Figure 1 were analyzed (see Table S1 for values). Each dot represents an individual fly (wing shape averaged over the right and left sides). Canonical variates are axes that maximize variation among groups (discrimination) relative to within group variation. Percentages indicate the amount of variance explained by the axes. The first axis clearly contrasts GOF (*da::Gal4>RCG76*) and control (+/+) flies. The difference between sexes is mostly detected on the second axis. Grey: +/+ females; black: +*/+* males; light green: GOF females; dark green: GOF males. B: Shape change along the first axis. The grey wing is the consensus wing computed from all wings (i.e. the grand mean shape); the green wing represents the shape change when moving from control to GOF flies.

We ubiquitously overexpressed *CycG* using the *daughterless* (*da*) driver. *da::Gal4/+* females were crossed with *UAS::mRFP-CycG (RCG76)/+* males as indicated in Material and Methods. *da>RCG76* flies, hereafter referred to as GOF for Gain of Function, were smaller and had smaller wings than control *+/+, da:Gal4/+* and *RCG76/+* siblings (wings around 15% smaller in both sexes; Table S1). Although wing venation pattern remained normal, wing shape was clearly affected by *CycG* overexpression, with a distally rounder shape and a parallel distal shift of both cross-veins (Figure 2, Table S1: significant genotype effect in the MANOVAs). Similar patterns of shape change were found in both sexes.

Wings of GOF flies exhibited a striking pattern of asymmetry detectable by sight as compared to wings of control siblings (Figure 1A). This asymmetry was not related to mRFP, as no such phenotype was observed in flies overexpressing *mRFP* using the same driver (*da>UAS::mRFP*) (data not shown). Reciprocally, flies overexpressing *CycG* without *mRFP* (i.e. *CG* transgenic lines) presented strong asymmetry (Figure S1C). To further check whether the asymmetry was specific to *CycG* overexpression, we used *da::Gal4* to overexpress other genes involved in cell growth or cell cycle regulation. None of the tested genes (*dS6K*, *Myc*, *CycD Cdk4*) produced any particular asymmetric pattern (data not shown). This also demonstrated that the asymmetry was not due to an asymmetric expression of the *da::Gal4* driver that would affect tissue growth differently in the left and right sides of individuals. Taken together, these results thus suggest that the observed asymmetry is an effect of *CycG* overexpression *per se*.

**Figure 3:**
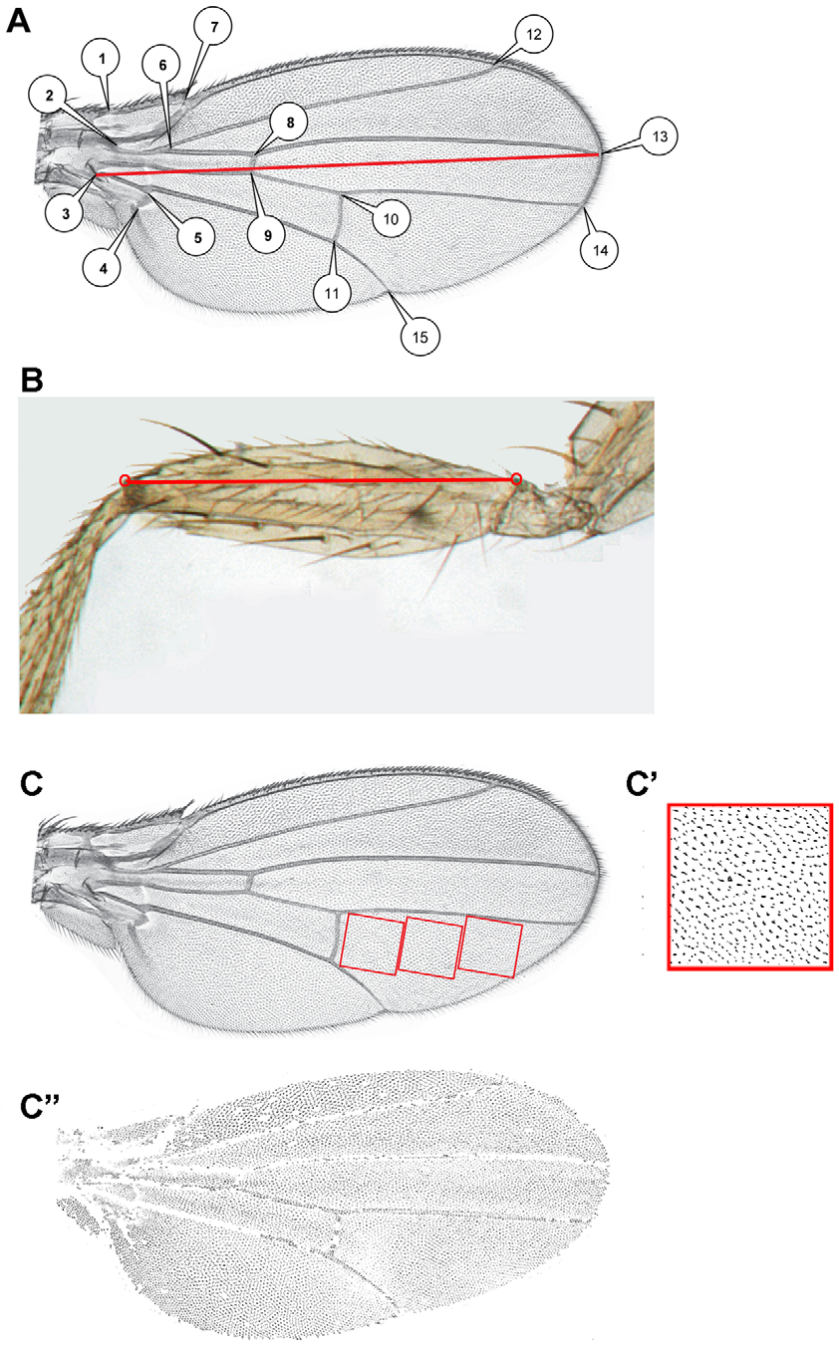
Acquisition of morphometric data. A: Position of the 15 landmarks digitized on the wings. The red line represents the measurement used as wing length for *sd>RCG76* flies. B: Landmarks used to measure femur length. C: Position of the standard areas used to estimate wing cell size. C′, C″: Typical thresholded pictures used to count cell size (C′) or total cell number (C″).

We therefore conducted a detailed analysis of wing size and shape asymmetry of GOF vs. control flies using replicated sets of landmark data [25], [26] (Figure 3A). Conspicuous biological asymmetries are generally either directional (i.e. one side, always the same, is larger in all individuals), or antisymmetric (i.e. all individuals are strongly asymmetric but equally frequently rightwards or leftwards). These asymmetries do not reflect developmental noise and thus cannot be used to assess developmental stability [26]. In contrast, developmental noise will randomly affect right and left sides generating the bilateral differences of random amplitude and directionality, known as fluctuating asymmetry (FA). The strong asymmetry observed in GOF flies turned out to be genuine FA of extreme amplitude (often reaching individual values of more than 10% of the trait value). The comparison of these GOF flies to control +/+ flies showed a 36.8-fold increase in wing size FA in females and a 38.9-fold increase in males (Figure 1B, Table S2). Wing shape asymmetry was also affected (7.6-fold increase in females and 10.2-fold increase in males as compared to +/+ controls; Figure 1B, Table S3).

### Replication of the *CycG*-induced FA effect in another genetic background

In order to investigate the potential effect of the genetic background, all transgenes, originally in a background marked by *w^1118^*, were introgressed into a new background marked by *yw^67c23^*. Microsatellite genotyping showed that the *w^1118^* background presented a low level of heterozygosity and that the *yw^67c23^* background was isogenic as far as can be detected (see Material and Methods). In the new background, wings of GOF flies were again smaller than the ones of +/+ controls (18% in both sexes; Table S1), and their shape was also altered (Table S1), shape changes being remarkably close to those found in the *w^1118^* background (compare Figure S1A, S1B to Figure 2A, 2B). The GOF flies exhibited extreme wing FA (26.9-fold increase in females and 48.2-fold increase in males for wing size, 9.2-fold increase in females and 11.1-fold increase in males for wing shape as compared to +/+ controls; Figure S2C; Tables S2, S3), clearly rejecting the hypothesis that FA increase was primarily an effect of the genetic background.

### *CycG* deregulation using different drivers increases FA

*CycG*-induced FA was further investigated using other drivers in the same isogenic *yw^67c23^* background. *Actin5C* (*Act*) served as an alternative ubiquitous driver. Wings of *Act>RCG76* flies were again smaller than the ones of control +/+ siblings (15% smaller for females and 20% for males, Table S1) and wing shape was altered as well (Table S1). Size FA doubled in both sexes (2.3-fold increase in females, 2.4-fold increase in males as compared to +/+ controls), and shape FA increased 9.8-fold in females and 2.8-fold in males (Tables S2, S3). A *scalloped* (*sd*) driver, *sd^29.1^*, hereafter called *sd::Gal4*, was further used to induce a more localized overexpression of *CycG* in wing imaginal discs. This driver is an insertion of a *P-Gal4* transgene that maps in the *scalloped* gene in the first large intron after the translational start site. It has been described as a weak hypomorph allele of *scalloped* [27]. Whereas wings of *sd::Gal4/+* flies have a wild type phenotype, wing margin of *sd>RCG76* flies was partially altered by notches (Figure S1E), suggesting that *CycG* interacted synergistically with *sd*. Wing centroid size and wing shape could thus not be analyzed, hence only wing length was measured (Figure 3A). Again, FA of *sd>RCG76* wings increased dramatically (6.8-fold for females and up to 47.9-fold for males) as compared to control +/+ siblings (Table S2). These results demonstrate that any *CycG* overexpression has a very strong effect on wing FA, although the driver can affect the intensity of the asymmetry response. qRT-PCRs showed that *CycG* overexpression driven by *Act* was weaker than the one driven by *da* (Figure S3), suggesting that the strength of the FA effect depends on the level of *CycG* expression.

### Reducing *CycG* expression by RNAi increases shape FA

We next examined the effects of a reduction in *CycG* expression on FA. Since no *CycG* mutant has been reported so far, the *UAS::dsCycG2* transgenic line was used to silence *CycG* by RNAi in the *yw^67c23^* background [21]. *CycG* was ubiquitously inactivated using the *da::Gal4* driver. In *da>dsCycG2* flies, hereafter referred to as LOF for Loss of Function, overall body size (including wing size) was not altered, in contrast to GOF flies (Table S1). Interestingly, wing shape was affected (Figure S2D, S2E; Table S1). Shape changes were similar in both sexes and were clearly different from those induced by *CycG* overexpression (compare Figure S2A, S2B to S2D, S2E).

Wing size FA increased significantly only in males (1.5-fold) as compared to +/+ siblings (Figure S2F, Table S2). However, wing shape FA increased by a factor of 3.7 in females and 2.3 in males (Figure S2F; Table S3), indicating that reducing *CycG* expression also impairs developmental stability.

### Patterns of *CycG*-induced wing shape asymmetry

**Figure 4:**
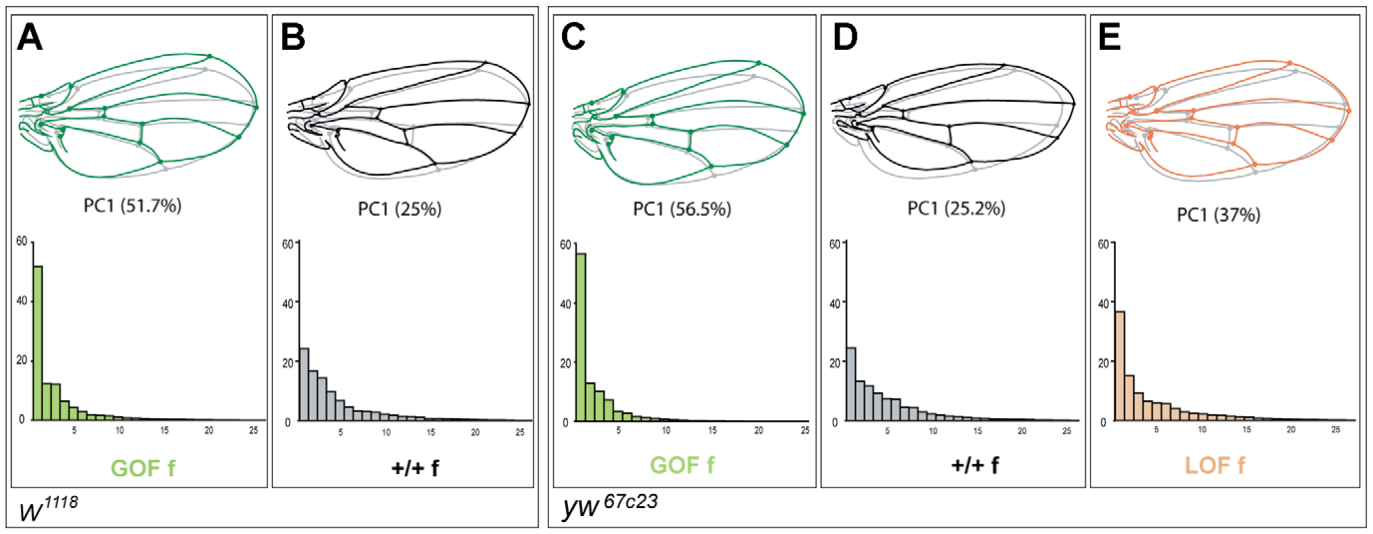
Patterns of wing shape FA in GOF, control and LOF female flies. Principal component analysis (PCA) of the wing shape FA matrices (note that since the PCAs were ran independently, eigenvalues are not directly comparable). GOF flies in the *w^1118^* and *yw^67c23^* backgrounds (same individuals as in Figure 1 and Figure S3A, S3B, S3C, respectively) and LOF flies in the *yw^67c23^* background (same individuals as in Figure S3D, S3E, S3F) were analyzed (see Table S4 for values). Only females are represented here. Top: Patterns of wing shape asymmetry associated with the first PC of the FA matrices. Grey shape: consensus wing computed from all wings; colored shape: shape asymmetry associated with the first principal component (PC). Bottom: Histograms of FA eigenvalues (i.e. amount of shape variance explained by each PC). Green: GOF flies (A, C); grey: control flies (B, D); orange: LOF flies (E).

FA occurs at low levels in any bilateral quantitative trait. Whether an increase in asymmetry is merely an amplification of the "normal" asymmetry or rather involves different processes is unknown. Simple traits, like size, vary in one dimension only and are therefore not amenable to address this question. Investigating wing shape asymmetry enables us to tackle this issue. If only amplification of normal asymmetry is involved, then the patterns of shape asymmetry should be unchanged. We computed the principal components of the shape asymmetry matrices for control and experimental flies, i.e. GOF flies in both *w^1118^* and *yw^67c23^* backgrounds and LOF flies in the *yw^67c23^* background (Figure 4). In contrast to +/+ siblings, shape FA matrices of GOF flies were clearly dominated by the first principal component (PC), indicating a strong structure of the FA effect along one dominant direction of shape change. This structure was less pronounced but also detected in LOF flies. Remarkably, while the wings of GOF and LOF flies differed in their mean shape, their patterns of shape FA were almost identical (Figure 4A, 4C, 4E; Table S4), as shown by the similarity of the FA PC1s (see Material and Methods). These patterns of shape FA were also significantly correlated with those of their +/+ siblings (Figure 4B, 4D; Table S4) despite some differences. In the *w^1118^* background, the patterns of shape FA between +/+ and GOF female flies did not even differ more than expected from sampling error only (i.e. the angle between their respective FA PC1s was smaller than between pairs of vectors derived from a distribution obtained by resampling within a single genotype; see Material and Methods; Table S4). This therefore suggests that the *CycG-*induced shape asymmetry mainly consists in an amplification of the dominant pattern of "normal" asymmetry.

### Effects of *CycG* overexpression on an other trait

We next asked whether the observed FA in flies deregulating *CycG* was restricted to wings or affected other body parts. To avoid a bias due to developmental correlation, we focused on a structure located on a different thoracic segment i.e. the first leg. First leg femurs of GOF flies previously analyzed for wing asymmetry (*da>RCG76* in the *yw^67c23^* background) or their +/+ siblings, were scored for length (Figure 3B). As expected from the reduced size of GOF flies, their femurs were shorter than those of control siblings (-14% in females and -16% in males). Consistently with wing analyses, *CycG* overexpression induced an increase in FA in both sexes (4.2-fold for females and 2.6-fold for males as compared to +/+ siblings; Table S5). Hence, the increase of asymmetry generated by deregulating *CycG* was not limited to wings but also affected other body parts. Noteworthy, we observed no individual correlation between wing FA and femur FA, i.e. the most asymmetric individuals for wings were not necessarily the most asymmetric for femurs, and *vice-versa*.

### Effects of *CycG* deregulation on cell size and cell number

**Figure 5:**
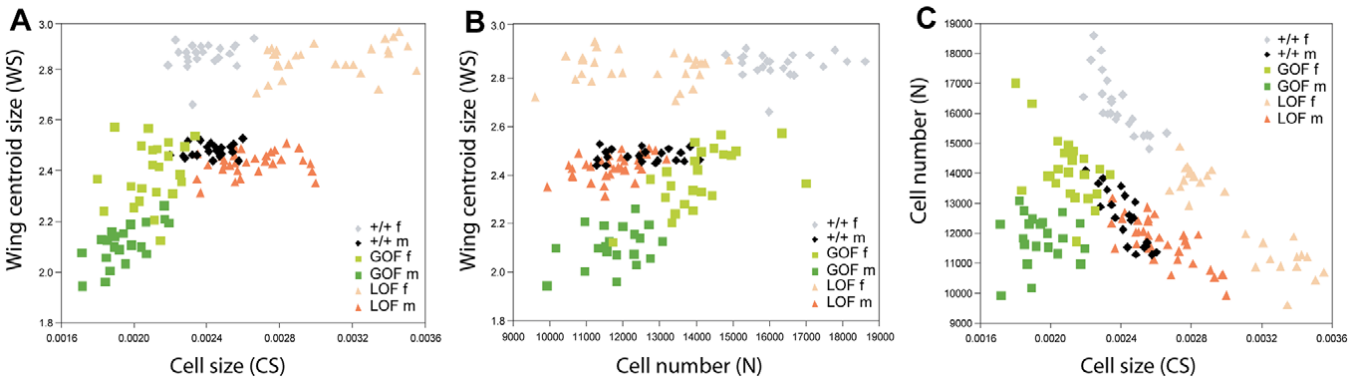
Relationship among wing size, cell size, and cell number. The progeny of crosses between *da::Gal4/+* females and *RCG76/+* males (GOF) and between *da::Gal4/+* females and *UAS::dsCycG2/+* males (LOF) in the same *yw^67c23^* background were analyzed (same individuals as in Figure S4). Each dot represents an individual fly (see Table S4 for values). A: Cell size (CS) vs. wing size (WS); B: Cell number (N) vs. wing size (WS). C: Cell size (CS) vs. cell number (N). Grey: *+/+* females; black: *+/+* males; light green: GOF females; dark green: GOF males; light orange: LOF females; dark orange: LOF males. Note that the tight negative relationship between cell size and cell number found in control *+/+* flies is altered in *CycG* deregulated flies. It is strongly affected in GOF female flies and lost in GOF males. While a negative correlation still holds for LOF flies, the strength of the relationship is altered (see text and Table S7).

To identify the cellular processes mediating the observed phenotypes, cell number and cell size were estimated on wings previously measured and scored for asymmetry (GOF and LOF flies, as well as their +/+ siblings, in the *yw^67c23^* background; Figure 3C, 3C″, 3C″). The overall effects of deregulating *CycG* are shown in Figure 5 and Table S6. For cell size, overexpression and inactivation of *CycG* had opposite effects: in both sexes, overexpression reduced cell size while inactivation increased it (Figure 5A; Table S6, top). For cell number, the effects were sex-specific: while *CycG* overexpression and inactivation both decreased cell number in females, no significant effect was detected in males (Figure 5B; Table S6, bottom).

Although wing size sexual dimorphism was stable across genotypes, females being typically larger than males (between 15 and 17% larger; Table S1), differences were found in the cellular basis of this dimorphism (Figure 5A, 5B; Table S6). In control flies, cell size was similar in males and females: sexual dimorphism was mainly due to cell number, the larger female wings bearing more cells that the smaller male ones. In GOF flies, both cell size and cell number differed between sexes, males having less cells of smaller size than females. In LOF flies, sexual dimorphism was mainly due to cell size, males having as many but smaller cells as females.

The smaller wing size of GOF flies was mainly explained by a decrease in cell size in both sexes as compared to +/+ siblings; cell number also decreased in females, while almost no difference was found in males (Figure 5A, 5B). Interestingly, in LOF flies, although wing size was not altered, both cell size and cell number were affected. Cells were bigger than those of controls in both sexes, although the difference was stronger in females. This effect was compensated by a reduction of cell number leading to a stable wing size (Figure 5A, 5B).

Remarkably, the relationship between cell size and cell number observed in wild type and LOF wings was strongly affected by *CycG* deregulation (Figure 5C, Table S6). Cell size and cell number were indeed tightly negatively correlated in control +/+ wings (+/+ females: r = -0.78***; +/+ males: r = -0.84***, see Table S6 for statistical comparisons). This correlation sharply decreased when *CycG* was overexpressed (GOF females: r = -0.52*; GOF males: r = -0.04, non significant, see Table S6 for statistical comparisons). This apparent uncoupling between the two cellular parameters was further evidenced when analyzing residuals of the regressions of cell size on cell number (Table S6). Their variance was indeed found significantly higher in GOF flies compared to controls. Interestingly, while the negative correlation still held for LOF flies (LOF females: r = -0.91***; LOF males: r = -0.77***; Table S7), the variance of the regression residuals also significantly increased relative to the controls, suggesting that the size/number relationship was altered as well when reducing *CycG* expression.

**Figure 6:**
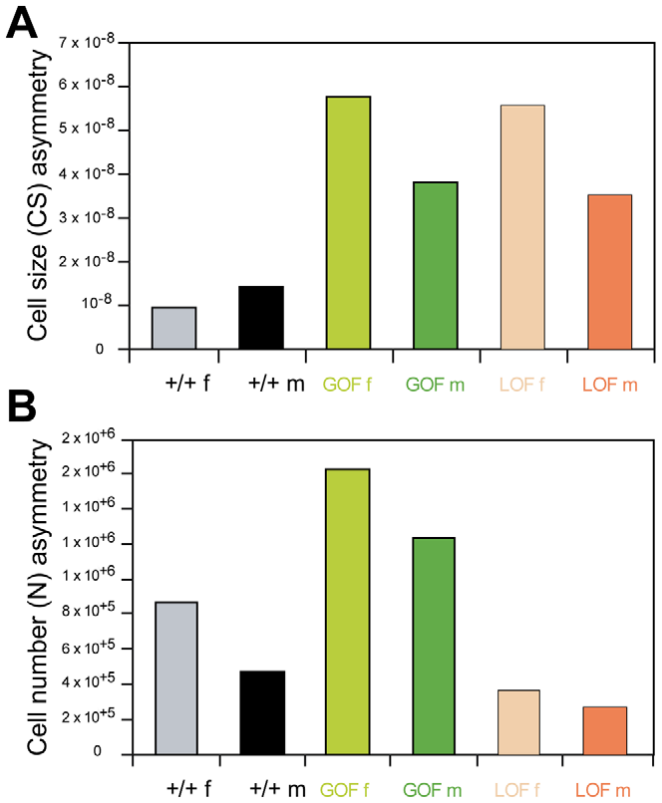
Fluctuating asymmetry of wing cell size and cell number. The same individuals as in Figure 5 were analyzed (see Table S7 for values). A: Cell size fluctuating asymmetry (FA4, see Material and Methods). B: Cell number fluctuating asymmetry (FA4). Grey: +/+ females; black: +*/+* males; light green: GOF females; dark green: GOF males; light orange: LOF females; dark orange: LOF males.

FA of cell size and cell number was measured to try to account for the extreme wing size asymmetries (Figure 6; Table S8). In both GOF and LOF flies, FA of cell size significantly increased relative to +/+ controls whereas FA of cell number did not vary. Furthermore, only cell size FA was found positively correlated with wing size FA across individuals (Table S8, bottom): flies strongly asymmetrical for wing size tended to be strongly asymmetrical for cell size (GOF females: r = 0.65**; GOF males: r = 0.54**) but not for cell number (GOF females: r = 0.11, non significant; GOF males: r = 0.27, non significant). Wing size asymmetry induced by *CycG* deregulation thus appeared to be predominantly generated by cell size asymmetry.

## Discussion

The *CycG* gene of *Drosophila melanogaster* encodes a cyclin involved in transcriptional regulation, cell growth and cell cycle [21], [22]. We report here that upregulation of *CycG* in a context where genetic and environmental variations were minimal induces extremely high levels of fluctuating asymmetry (FA) in several traits, suggesting that Cyclin G is a major factor of developmental stability.

### Deregulating *CycG* alters cell growth and the compensation between cell proliferation and cell growth

Cell growth is markedly downregulated by *CycG* as *CycG* inactivation increases adult wing cell size while *CycG* overexpression reduces it. In wing imaginal discs, however, although cell size is also reduced by *CycG* overexpression, inactivation of *CycG* only induces a slight increase in cell size [22]. This suggests that in flies where *CycG* is inactivated, extra cell growth occurs during post-larval stages. Furthermore, *CycG* impairs not only cell growth but also cell proliferation. Indeed, both inactivation and overexpression lead to a reduction in cell number in females. The fact that wings of flies where *CycG* was inactivated reach a size comparable to that of wild type flies suggests that cell growth compensates for lack of cell proliferation.

A tight negative correlation between cell size and cell number is observed in control flies suggesting that wing size stability is ensured by compensation between cell proliferation and cell growth. This has also been observed in natural populations, where cell size and cell number tend to show negative covariance [28], [29]. In addition, genetic manipulation of cell size using *cdc2* mutants [30], *dMyc* mutants [31] or deregulation of cell cycle regulators [32] confirms that cell growth and proliferation can compensate each other to reach normal organ size. Hence, the final size of the wing seems to be determined by a compensatory mechanism between cell size and cell number. This mechanism is deeply impaired in *CycG* overexpressing flies. Remarkably, although the negative correlation is significant in LOF flies, the variance of the regression residuals presents a sharp increase relative to the controls, indicating a loosening in the relationship. Altogether these results suggest that deregulating *CycG* alters the link between cell growth and proliferation. This in turn suggests that compensation between cell growth and division is one key factor in maintaining wing size – and thus wing developmental stability – and that *CycG* is critical for ensuring this compensation.

### Deregulating *CycG* alters the cellular basis of wing sexual size dimorphism

In natural populations, differences in wing size between sexes have been suggested to involve both cell size and cell number [29], [33]. In our control isogenic lines though, wing sexual size dimorphism was only due to cell number, cell size being strikingly similar in both sexes. This suggests that adaptation to laboratory conditions or genetic drift might affect the cellular basis of sexual size dimorphism.

Cell size is affected similarly in both sexes when manipulating *CycG* expression. In contrast, the effects on cell number are different between sexes: while no effect is detectable in males, both GOF and LOF females have fewer cells than the controls. During the pupal stage, wing cells undergo two rounds of division [34]. As G-type cyclins are known to be important in terminally differentiated cells [35], it is tempting to speculate that these last divisions are differentially regulated in males and females and are controlled by Cyclin G. The last divisions in the pupal wing might be a crucial determinant of the sexual dimorphism of wing size.

### Deregulating *CycG* alters wing shape

The altered wing shape in both GOF and LOF flies suggests that the ubiquitous *da* deregulation of *CycG* across the wing blade induced heterogeneous effects on cell size and cell number, possibly reflecting an interference with morphogens driving wing growth. Nevertheless, the changes in mean wing shape found in GOF flies are clearly different from those in LOF flies. As the patterns of shape change remained different in LOF and GOF flies after correcting for size – only the GOF flies were smaller than the controls, we could rule out the hypothesis of a simple allometric effect (Figure S4). A detailed mapping of the cellular effects on the wing would be needed to relate the shape changes to morphogenetic processes. These results are nevertheless consistent with QTL analyses showing that wing shape is regulated at least partly independently of wing size [36]. Wing veins are important determinants of wing shape [37] and wing shape has notably been shown to be tightly associated with the *Egf receptor* locus that controls the amount of vein material [36], [38], [39]. Interestingly, *torpedo*, a mutant of the EGF Receptor, shows similar abdomen cuticle defects [40] than those observed in *CycG* LOF flies [20]. Thus, *CycG* might also interact with the *Egf receptor* in the wing imaginal disc to control vein specification and wing shape.

### Upregulating *CycG* increases size FA

The amplitude of the asymmetry effect observed when overexpressing *CycG* is particularly dramatic, and such an amplitude is usually associated with directional asymmetry or antisymmetry, the two forms of conspicuous asymmetry. Similar levels of FA have – to our knowledge – never been reported. Comparatively, a study using deletions covering most of the *Drosophila* genome detected a maximum increase of 7-fold in size FA [13] (Breuker, personal communication).

The very low level of genetic variation and the carefully controlled environmental conditions ensured that this effect was due to developmental noise and was not confounded with genetic or environmental variation for directional asymmetry ([41], [42]; see Material and Methods).

That wing size and shape as well as femurs of the first leg are affected demonstrates that, although the strength of the effect on FA may vary across body parts, this effect is not restricted to a single trait *(*i.e. the wing) or a specific segment. This result is particularly important since the only previously known cases of individual genes altering FA were trait-specific [16]. Although wings and legs are both thoracic appendages with partly similar developmental networks, such a common FA effect suggests that the cellular processes altered by deregulation of *CycG* are likely common to many traits. It also provides some support to the hypothesis of an organism-wide source of developmental noise, and indirectly it suggests the existence of organism-wide stabilizing processes, a very contentious issue [43].

Some preliminary tests on bristle traits nevertheless suggest that bristle number FA is not affected by *CycG* deregulation. This is in agreement with previous studies suggesting that meristic and metric trait variation could be controlled *via* different processes [10], [44].

### Deregulating *CycG* increases shape FA

Whereas mean wing shape is affected differently in LOF and GOF flies, the patterns of shape variation around these different means, and specifically those of shape FA, are strikingly similar. This similarity can be interpreted in different ways. First, it might indicate that stochastic variation is constrained along a limited set of directions of shape change, consistent with the view of a wing as an integrated system [45]. Alternatively, such similarity of patterns might reflect a similarity of processes. Although mean wing shape is affected differently when increasing or reducing *CycG* expression, it is conceivable that shifting *CycG* expression level away from its normal value might destabilize development in similar ways, generating these similar patterns of shape FA. Comparable - although not identical - patterns of wing shape FA were found in control flies, suggesting that similar processes are involved in generating FA in wild type and *CycG* deregulated flies. This again supports the view that *CycG* plays an important role in developmental stability.

### Genetics and regulation of developmental stability

*CycG* thus appears as a serious candidate for the genetic control of developmental stability, and further studies should examine its role in FA amplitude differences across natural populations or samples submitted to various environmental treatments. Can we reconcile the reported lack of additive genetic variation for FA [14] with the putative existence of (a) major gene(s) altering FA? It is likely that *CycG* is involved in a genetic network regulating cell growth, and possibly cell proliferation [22], where it might act as a hub, as the high FA induced by overexpression suggests. Such a function, likely involving various pleiotropic effects and epistatic interactions, might be under strong selection, possibly leading to the elimination of any variation.

It is also conceivable that subtle variation in *CycG* may occur with only small effects on FA. In particular, *CycG* overexpression triggered by transgenic constructs is likely of larger magnitude compared to the effects of natural variation. Investigation of natural variation in *CycG* sequence and expression across populations differing in their degree of FA would provide some insight on this question. Investigating genes interacting with *CycG* would also improve our understanding of its link with organ size stochastic variation.

It was recently suggested that the ability of organs to reach a stereotypical size would depend on the competition among populations of growing cells [46]. In given developmental conditions (e.g. during the last cell divisions in the pupal wing blade), Cyclin G intracellular concentration might somehow trigger cell division. Pushing this concentration away from its usual value might interfere with the process by which cells identify the appropriate stage of growth for division, potentially generating stochasticity in cell size and decoupling cell growth and division. This might in turn compromise the normal pattern of cellular competition, causing random variation in organ size.

The extreme FA reported in this paper was generated by deregulating expression of a single gene. Consequently, the above hypothetical scenario focuses on the role of a single protein on the generation of random variation at the cellular level, but it does not preclude the existence of diverse processes working at various biological scales [47].

Our results do not necessarily mean that *CycG* is a gene *for* developmental stability, but they clearly show, by the strength of its effect on cell size variation, that *CycG* normal expression is required for the formation of symmetrical flies.

## Materials and Methods

### Fly strains

*UAS::mRFP-CycG* and *UAS::CycG* lines (respectively referred to as *RCG* and *CG*), containing the full-length *CycG* cDNA, were established by standard transformation [22]. The previously described *UAS::dsCycG2* line was used to downregulate *CycG* by RNAi [21]. *CycG* overexpression and downregulation were carried out using either ubiquitous Gal4 drivers *daughterless* (*da::Gal4*) or *Actin5C* (*Act::Gal4*) (NP3121, DGRC Kyoto), or the tissue-specific Gal4 driver *scalloped sd^29.1^ (*called *sd::Gal4)* (BL-8609). All these transgenic lines display promoter-independent *mini-white* expression.

Here, we present the results for one of the *RCG* lines only (*RCG76*), but similar effects were found with other *RCG* lines and *CG* lines (Figure S1). *UAS::mRFP* (BL-7118), *UAS::dS6K* [48], *UAS::Myc* (BL-9674), *UAS::CycD* [49] and *UAS::Cdk4* [50] were used as control lines.

Strains were maintained and crossed on standard medium at 25°C. For all crosses, 5 females heterozygous for a Gal4 driver were mated with 5 males heterozygous for an UAS transgene; they were transferred in a new vial every 24 h.

### Genetic background

The first analyses were performed using a genetic background marked by *w^1118^* i.e. the original background of all the transgenes. Trangenes were then introgressed into a new background marked by *yw^67c23^* and followed by eye-color. The *yw^67c23^* line was submitted to 10 rounds of isogenization prior to transgene introgression. Males *w^1118^/Y; da::Gal4* were crossed with *yw^67c23^* isogenic females. The resulting *yw^67c23^/Y; da::Gal4/+* males were individually crossed with isogenized *yw^67c23^* females. A first round of isogenization was then performed by individually crossing *yw^67c23^; da::Gal4/+* females with isogenic *yw^67c23^/Y* males. Females *yw^67c23^; da::Gal4/+* were recovered and individually crossed with isogenic *yw^67c23^/Y* males for a second round of isogenization. *yw^67c23^Act::Gal4* females were crossed with isogenized *yw^67c23^* males. *yw^67c23^Act::Gal4/yw^67c23^* females were then individually crossed with isogenized *yw^67c23^* males for a first round of isogenization. The same scheme was adopted for *yw^67c23^sd::Gal4* females. After ten rounds of isogenization, one single isogenic line was kept for each transgene.

Isogenicity of the *da::Gal4* and *RCG76* lines was assessed by analyzing 19 microsatellite markers distributed over the 4 chromosomes in 5 individual females. It revealed a single polymorphic locus for *w^1118^* females. However, the *w^1118^*; *da::Gal4* and *w^1118^*; *RCG76* females presented polymorphism at 7 and 2 additional loci, respectively. The *yw^67c23^* line was checked for the same 19 loci, revealing no polymorphism whereas *yw^67c23^; da::Gal4* and *yw^67c23^; RCG76* females each presented polymorphism at only 3 loci.

### Morphometrics

#### Acquisition

Right and left wings of the progeny from all crosses were mounted on slides, dorsal side up, and photographed using an Imasys uEye digital camera mounted on a Leica DMRB microscope. Legs of the progeny from the cross between *da::Gal4/+* and *RCG76/+* in the *yw^67c23^* genetic background were mounted on slides and photographed using a microscope equipped with a Nikon DXM 1200 camera. 15 landmarks per wing were digitized (Figure 3A). All measures were performed using Image J.

#### Size

Log of the centroid size was used as a size variable for the wing (i.e. the square root of the sum of the squared distances from each landmark to the corresponding configuration's centroid [23], [24]). In *sd>RCG76* flies, wing length was measured as the distance between landmark 3 and landmark 13 (Figure 3A). Length of the first leg femur was measured in arbitrary units as the distance between two landmarks as shown in Figure 3B.

#### Shape

Generalized least squares Procrustes superimposition was used to extract shape variation from the landmark data [23], [24]. In order to avoid problems related to loss of dimensions due to superimposition, a principal component analysis (PCA) was applied to the Procrustes coordinates (i.e. the coordinates after superimposition) and the non-zero PC scores were used as shape variables in all subsequent shape analyses.

#### Allometry

Allometry was investigated applying a multivariate regression of the PC scores on centroid size and using the residuals as allometry-free shape variables (e.g. [51]). Discriminant analyses ran before and after the regression were compared to assess the impact of allometry on wing shape changes among genotypes (Figure S4).

#### Cell number and cell size

A 256×256 pixel area, corforesponding to a 0.07 mm^2^ square, was displayed in the intervein region between vein 4 and vein 5 just behind the posterior cross-vein (Figure 3C). Thresholding was performed to keep epidermal hairs of the dorsal cell layer only using Image J software (Figure 3C′). Cells were automatically counted considering that each cell carries one epidermal hair. To account for potential heterogeneity in cell density in the wing, the area was then shifted twice along the proximo-distal axis and cells were counted again. As no significant difference among the three sampled regions was found, the number of cells counted over these three regions for each wing was averaged. Cell size was computed as one divided by the number of cells in the sampled area. The total number of cells in the dorsal cell layer of the wing was obtained applying the same thresholding to the whole wing. This thresholding mostly excluded cells located on the veins and the wing margin (Figure 3C″). We therefore underestimated the real cell numbers, but this bias was likely stable across individuals and genotypes.

The relationship between cell size and cell number was analyzed as follows.

We first computed the Pearson correlation coefficient (r) for each genotype. These correlations were then compared among genotypes using Fisher r to Z transformation. To investigate more accurately the strength of the relationship between the two cellular parameters, we ran a regression model (cell size over cell number) on each genotype and computed the variance of the residuals: the stronger the association, the lower the variance. This variance was then compared across genotypes using a Levene test followed by pairwise F tests.

### FA analysis

#### Antisymmetry

We did not detect antisymmetry in any trait investigated: no evidence for bimodality was found in right minus left distributions of size-related traits (not shown); for wing shape data, visual inspection of right-left shape vectors did not suggest occurrence of any clustering, as would be expected for antisymmetric traits.

#### Measurement error

Measurement error (ME) is of critical importance when analyzing fluctuating asymmetry [26]. To quantify ME, both sides were measured several times. All flies used in the overexpression experiments using *da* were digitized twice for wing size and shape, and three times for femurs. As measurement error was shown to be negligible relative to true FA (interaction MS relative to error MS in Tables S2, S3, S4), a single measurement session was used in subsequent experiments (i.e. the ones involving *Act* and *sd*). Conventional two-way mixed model ANOVAs were applied to size data (centroid size, wing length and femur length) and Procrustes ANOVAs to wing shape data, using Individual (random), Side (fixed) and their interaction as effects. The details of this procedure can be found in [25], [26]. In addition to the estimation of ME, this ANOVA allows testing for the occurrence of directional asymmetry. For size, the side effect was never statistically significant in GOF and LOF flies (see "side" effect P-values on Table S2) demonstrating that true FA rather than directional asymmetry is responsible for the observed asymmetry pattern. Wing shape directional asymmetry was detected in some cases (see "side" effect P-values on Table S3). Estimators of FA corrected for ME and directional asymmetry (FA10) were then derived from these ANOVAs (FA10 following the standard terminology by Palmer and Strobeck [26]). When only one measurement was available, variance of (R-L) was used as an FA index (FA4, [26]). The relationship between size and asymmetry was examined to test the occurrence of allometric effect on FA. Such an effect was detected among genotypes but is mostly attributable to the fact that flies overexpressing *CycG* are smaller than the controls. No significant allometric effect was detected within genotypes.

Comparisons of FA values among genotypes were done using standard F-tests. P-values were adjusted using the Holm procedure [52] each time the analyses involved multiple comparisons.

#### Individual variation for directional asymmetry

Even when the average asymmetry is zero, genetic or environmental variation for directional asymmetry can occur and inflate the apparent level of fluctuating asymmetry, thereby impeding its use as an estimator of developmental stability (e.g. [42]). Genetic variation for directional asymmetry has been reported to be very low in most cases and artificial selection experiments in Drosophila have failed to generate a significant increase in asymmetry (reviewed in [41]). Some studies have nevertheless reported significant genetic variation for directional asymmetry (see [53] for a very strong effect in Drosophila interspecific hybrids). We could rule out the hypothesis of such an individual variation for directional asymmetry as the genetic variation was reduced to a minimum by the inbreeding procedure and by raising the flies in a carefully controlled environment.

#### Patterns of wing shape FA

Patterns of shape asymmetry were investigated following Klingenberg and McIntyre [25]. For each genotype and sex, we first computed the matrix of shape FA as the covariance matrix of the individual*side effect corrected for measurement error. We then ran a Principal Component Analysis (PCA) on this matrix. We examined the distribution of the eigenvalues [decreasing amount of variation accounted for by the successive principal components (PCs)]. Note that since the PCAs were ran independently, there is no one-to-one correspondence between PCs. Comparing individual eigenvalues across genotypes is thus non informative. In contrast, the shape of the whole distribution indicates whether shape FA is concentrated along one specific direction or rather is distributed over many directions. Using multivariate regression, we then displayed graphically the dominant pattern of shape FA as the shape change associated with the first FA PC. This allowed a direct visual comparison of the patterns of shape FA among genotypes.

The significance of the correlation of shape FA patterns among genotypes was then tested as follows. As the angles formed by pairs of FA PCs estimate their similarity, we first compared them to a null distribution of angles formed by pairs of 26-dimensional random vectors. This allowed testing whether the recorded patterns of FA were more similar than expected from random variation. We then examined the alternative null hypothesis of identical FA vectors. To test whether the vectors differed more than expected from sampling error alone, we compared them to a null distribution of angles obtained from a bootstrap procedure resampling individual observations within genotype.

#### FA of cell size and cell number

Fluctuating asymmetry of cell size and cell number was computed for each genotype as the variance of (R-L) values (FA4).

Morphometric and statistical analyses were conducted using R version 2.6.2 [54] and the MorphoJ package [55].

## Supporting Information

Figure S1 Superimposition of wings. In each case, photos of the two wings of the same individual were superimposed: in red the left wing and in green the right wing. A, B and C: Individuals overexpressing *CycG* ubiquitously under control of the *da::Gal4* driver using different *RCG* lines (*RCG23.3, RCG69*) or CG line (*CG2.1*). D and E: Individuals overexpressing *CycG* (*RCG76* line) under control of different drivers (*Act::Gal4* and *sd::Gal4*). F: Individual RNAi-inactivating *CycG* ubiquitously with the *da::Gal4* driver (LOF).
(TIF)

Figure S2 Effects of *CycG* deregulation in a *yw^67c23^* background. A, B, and C: *CycG* overexpression; D, E and F: *CycG* inactivation. A and D: Discriminant analysis of the wing shape data. Each dot represents an individual fly (wing shape averaged over the right and left sides). GOF flies and LOF flies are completely discriminated from control flies, respectively on the first and second axes. Grey: +/+ females; black: +*/+* males; light green: GOF females; dark green: GOF males; light orange: LOF females; dark orange: LOF males; B and E: Shape change along the first axis; GOF wings (B) and LOF wings (E) (note that axes are inverted relative to the GOF experiment, due to a lesser amplitude of shape change among genotypes). The grey wing is the consensus wing computed from all wings (i.e. the grand mean shape); the colored wing represents the shape change when moving from +/+ control to GOF or LOF wings. C and F: Effect of *CycG* deregulation on wing size FA (open bars) and shape FA (dashed bars). Note that FA values reported on C and F are not directly comparable: FA10 indice was used in C, and FA4 in F (see Material and Methods).
(TIF)

Figure S3 Overexpression and downregulation of *CycG*. Total RNA was extracted from third instar larvae using the RNeasy kit (Qiagen). Real-time PCR was performed in triplicate using Taqman® Gene Expression Assays (Dm02151951_m1 CycG, Applied Biosystems) on a ABI prism 7700 detection system. Results were normalized against *Gapdh1* (Dm01843827_s1, Applied Biosystems) using the 2exp^-ΔΔCt^ method.
(TIF)

Figure S4 Impact of allometry on mean wing shape discrimination (*yw^67c2^* background). A: Discriminant analysis applied to the shape variables (i.e. non null PC scores; grouping factor: genotype*sex). B: Discriminant analysis applied to the residuals of a multivariate regression of size on shape variables. Grey: +/+ females; black: +*/+* males; light green: GOF females; dark green: GOF males; light orange: LOF females; dark orange: LOF males.
(TIF)

Table S1 Effects of *CycG* deregulation on mean wing size and shape. Mean centroid size values and standard deviations (Sd) are provided. Results of the ANOVAs on centroid size and MANOVAs on the PC scores (genotype and sex as fixed factors). GOF =  gain of function; LOF =  loss of function; +/+  =  controls; f =  females; m =  males. Sd =  standard deviation; Df =  degrees of freedom; SS =  sum of squares; MS =  mean squares; F =  Fisher's F value; Pillai =  Pillai's Trace; Df den =  denominator's degrees of freedom; Df num =  numerator's degrees of freedom; * = p<0.05; ** = p<0.01; *** = p<0.001; ns =  non significant.
(DOC)

Table S2 Wing size FA. Results of the two-way mixed model ANOVAs on centroid size (individual  =  random; side  =  fixed). The tests are presented for the three control genotypes in the GOF experiments using *da* as driver (see Material and Methods). FA10 and FA4 are two FA indices (see Material and Methods). FA effect refers to the ratio of the GOF or LOF FA value over the one of the corresponding *+/+* genotype. Df =  degrees of freedom; MS =  mean squares; F =  Fisher's F value. MS and FA10 values are multiplied by 10^5^.
(DOC)

Table S3 Wing shape FA. Results of the Procrustes ANOVAs (individual and side as main effects). MS and FA10 values are multiplied by 10^7^. Df =  degrees of freedom; MS =  mean squares; F =  Fisher's F value.
(DOC)

Table S4 Comparison of patterns of shape FA (females). Correlation of PC1s of FA matrices for all genotypes as measured by the angles among PCs. In brackets are the associated P-values. Top: H0 =  the null hypothesis is that the angles between FA PCs are not different from those between pairs of random vectors (10000 random 26 dimensional vectors). A significant effect means that the correlation is stronger than expected from chance only. The P-value is computed as [1 -(number of random angles larger than the observed one)]/10000. Bottom: H0 =  the null hypothesis is that the angles between FA PCs are not larger from those between pairs of vectors differing only by the sampling error. Statistical significance is tested against a null distribution of vectors derived from a within genotype boostrap procedure (x10000). A non significant effect means that the vectors are as strongly correlated as vectors differing only by sampling error (i.e. they are almost identical). The P-value is computed as [(number of bootstrapped angles larger than the observed one)]/10000. * = p <0.05; ** = p<0.01; *** = p<0.001, ns =  non significant.
(DOC)

Table S5 Femur length FA. Results of the two-way mixed model ANOVAs on femur length (individual  =  random; side  =  fixed). Df =  degrees of freedom; MS =  mean squares; F =  Fisher's F value.
(DOC)

Table S6 Effects on mean cell size and cell number. Mean wing cell size and cell number and standard deviations (Sd) are provided. Note that cell size is in arbitrary unit, being computed as one divided by the number of cells counted in the standardized area (see Figure 3C′). Cell number strongly underestimate the real cell number in the wing: only one side of the wing was considered (dorsal); the wing basis was not included in the analysis; cells on - or at the direct vicinity of - veins were excluded due to the image thresholding (see Figure 3C″. This underestimation is likely similar across individuals and genotypes and should thus not affect the results. ANOVAs (genotype and sex as main fixed effects) are presented with post hoc Tukey HSD test. Df =  degrees of freedom; SS =  sums of squares; MS =  mean squares; F =  Fisher's F-test value; Diff =  Difference in the observed means; lwr and upr  =  lower and upper limits of the interval range for each comparison; p-adj  =  adjusted P-value; * = p <0.05; *** = p<0.001, ns =  non significant.
(DOC)

Table S7 Covariation between cell size and cell number. Top: Pearson 's correlation coefficients (r) were computed for each genotypes. They were then compared statistically after applying a Fisher r to Z transformation. Bottom: Analysis of the residuals of regressions of cell size over cell number for each genotype and sex. Statistical significance of variance differences are tested using standard F tests. Z =  Fisher Z values; F =  Fisher F; p =  P-values.
(DOC)

Table S8 Effects on cell size and cell number FA. FA values for each genotype and sex is provided, as well as the statistical tests of comparison with the controls (F tests). The correlation between cell size and cell number FA with wing size FA were computed. Both Pearson parametric correlation coefficient and Spearman non parametric correlation were computed. Only Pearson's r values are shown with the corresponding test of statistical significance, as both tests provided very similar results. Df = degrees of freedom; MS = mean squares; F =  Fisher's F value; * = p<0.05; ** = p<0.01; ***  = p<0.001; ns =  non significant.
(DOC)
