# Supplementary figures and images for: Developmental Stability: A Major Role for Cyclin G in Drosophila melanogaster
Source: PLoS Genet. 2011 Oct 6;7(10):e1002314. doi: 10.1371/journal.pgen.1002314 (PMC3188557; doi:10.1371/journal.pgen.1002314)

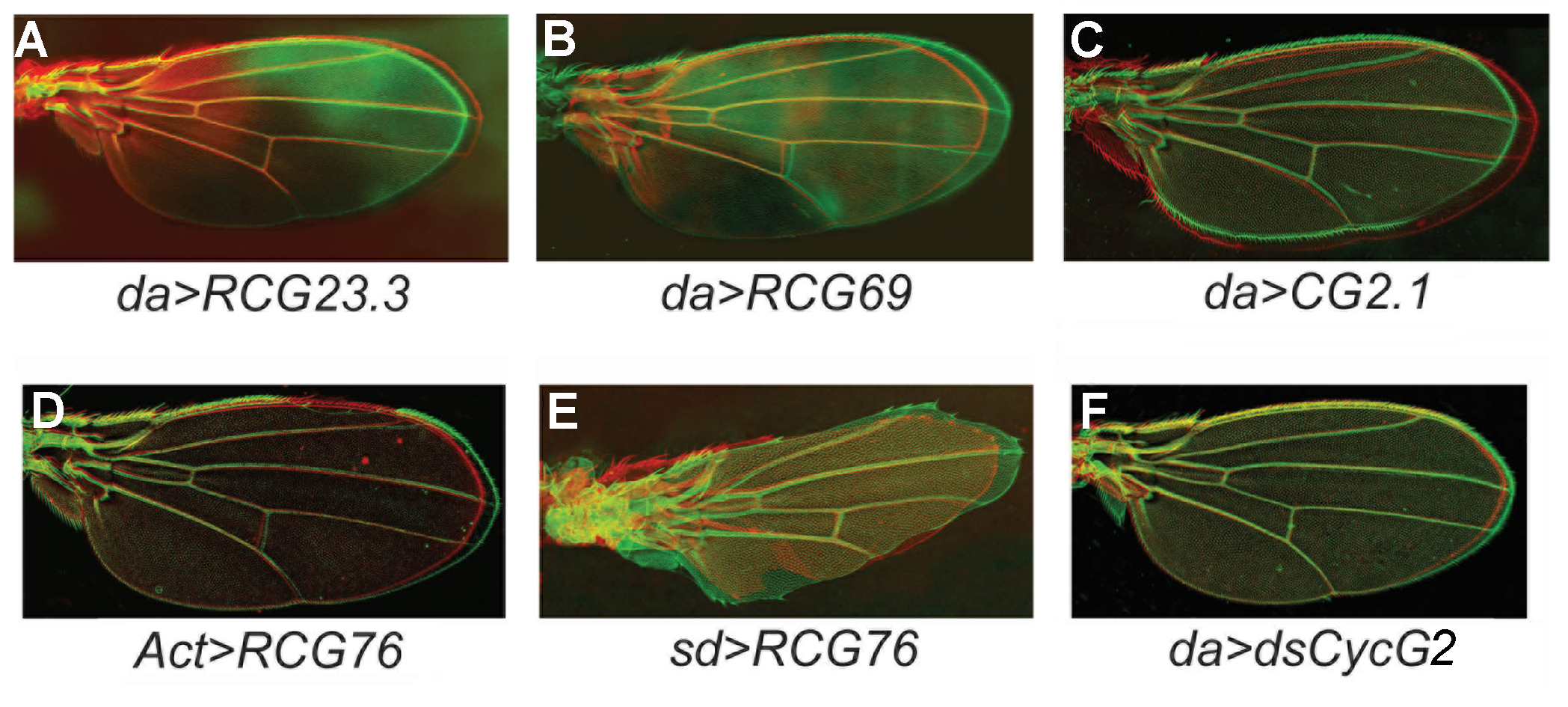

Supplement: Figure S1 — Superimposition of wings. In each case, photos of the two wings of the same individual were superimposed: in red the left wing and in green the right wing. A, B and C: Individuals overexpressing CycG ubiquitously under control of the da::Gal4 driver using different RCG lines (RCG23.3, RCG69) or CG line (CG2.1). D and E: Individuals overexpressing CycG (RCG76 line) under control of different drivers (Act::Gal4 and sd::Gal4). F: Individual RNAi-inactivating CycG ubiquitously with the da::Gal4 driver (LOF). (TIF) [file pgen.1002314.s001.tif]

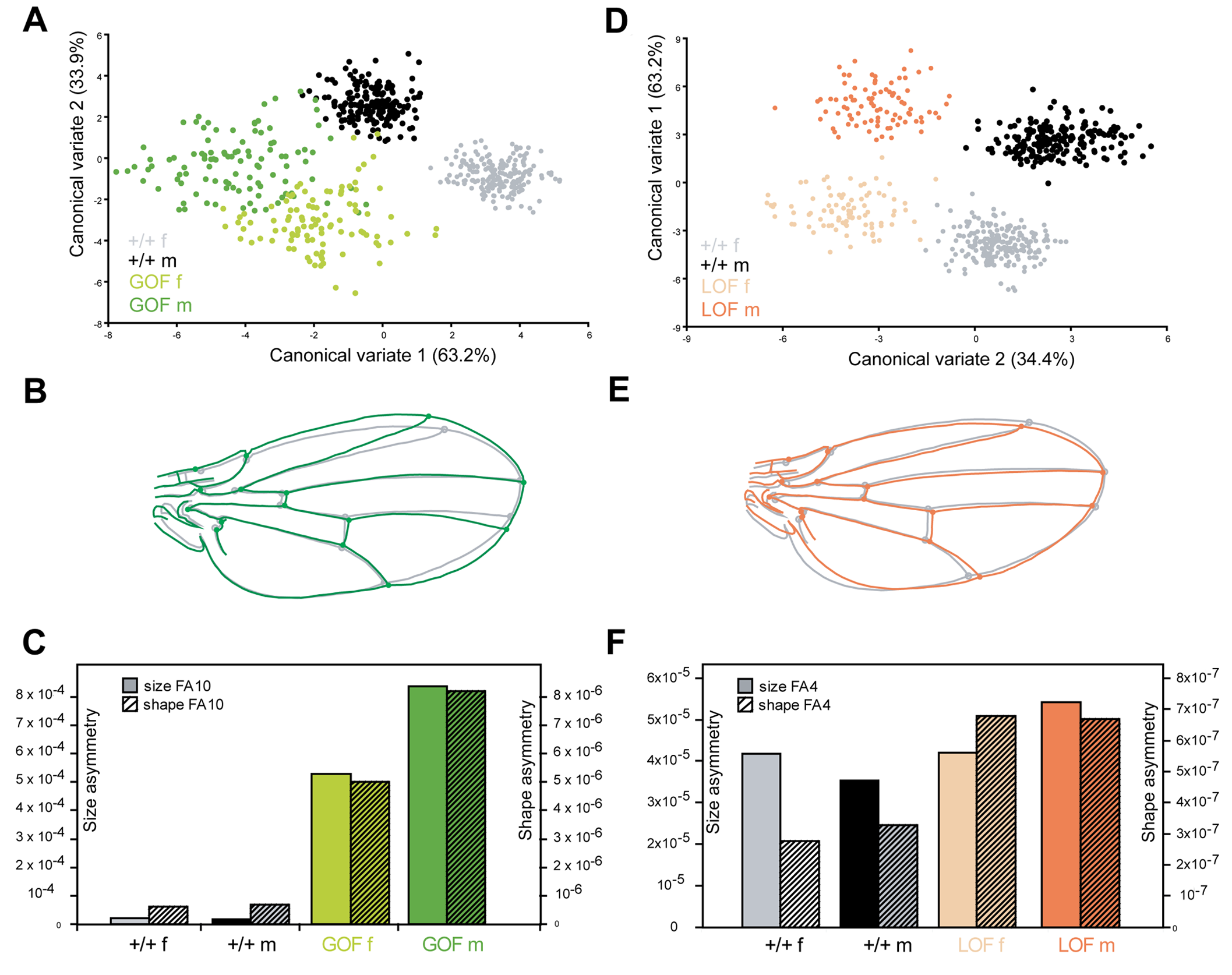

Supplement: Figure S2 — Effects of CycG deregulation in a yw67c23 background. A, B, and C: CycG overexpression; D, E and F: CycG inactivation. A and D: Discriminant analysis of the wing shape data. Each dot represents an individual fly (wing shape averaged over the right and left sides). GOF flies and LOF flies are completely discriminated from control flies, respectively on the first and second axes. Grey: +/+ females; black: +/+ males; light green: GOF females; dark green: GOF males; light orange: LOF females; dark orange: LOF males; B and E: Shape change along the first axis; GOF wings (B) and LOF wings (E) (note that axes are inverted relative to the GOF experiment, due to a lesser amplitude of shape change among genotypes). The grey wing is the consensus wing computed from all wings (i.e. the grand mean shape); the colored wing represents the shape change when moving from +/+ control to GOF or LOF wings. C and F: Effect of CycG deregulation on wing size FA (open bars) and shape FA (dashed bars). Note that FA values reported on C and F are not directly comparable: FA10 indice was used in C, and FA4 in F (see Material and Methods). (TIF) [file pgen.1002314.s002.tif]

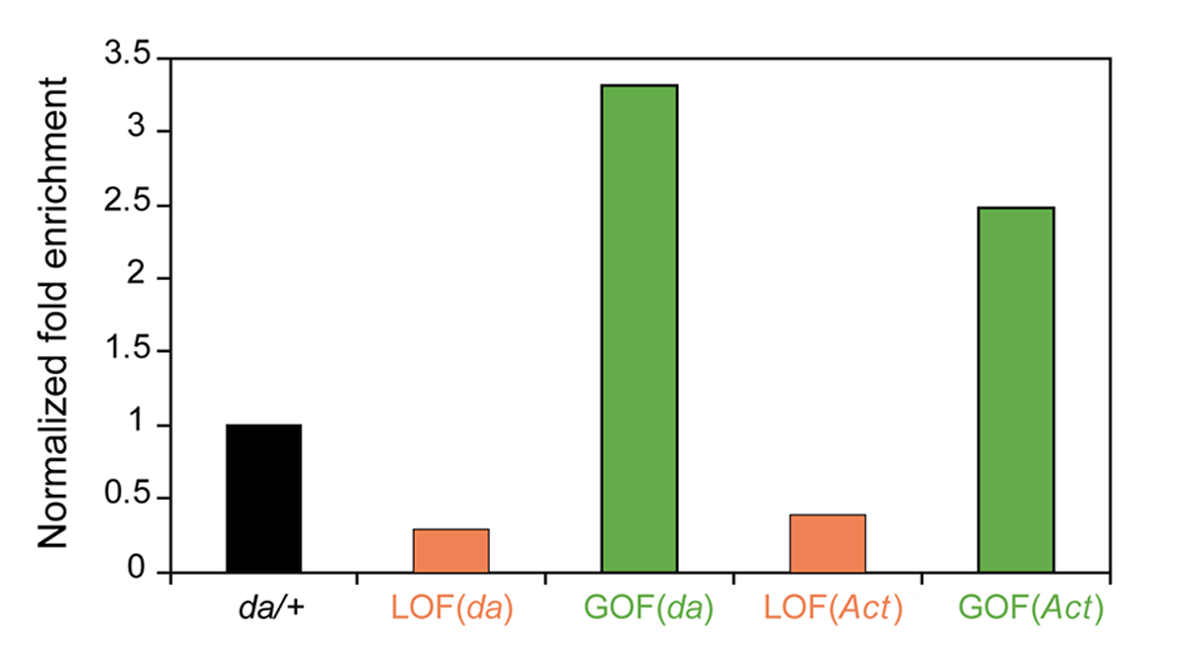

Supplement: Figure S3 — Overexpression and downregulation of CycG. Total RNA was extracted from third instar larvae using the RNeasy kit (Qiagen). Real-time PCR was performed in triplicate using Taqman® Gene Expression Assays (Dm02151951_m1 CycG, Applied Biosystems) on a ABI prism 7700 detection system. Results were normalized against Gapdh1 (Dm01843827_s1, Applied Biosystems) using the 2exp-ΔΔCt method. (TIF) [file pgen.1002314.s003.tif]

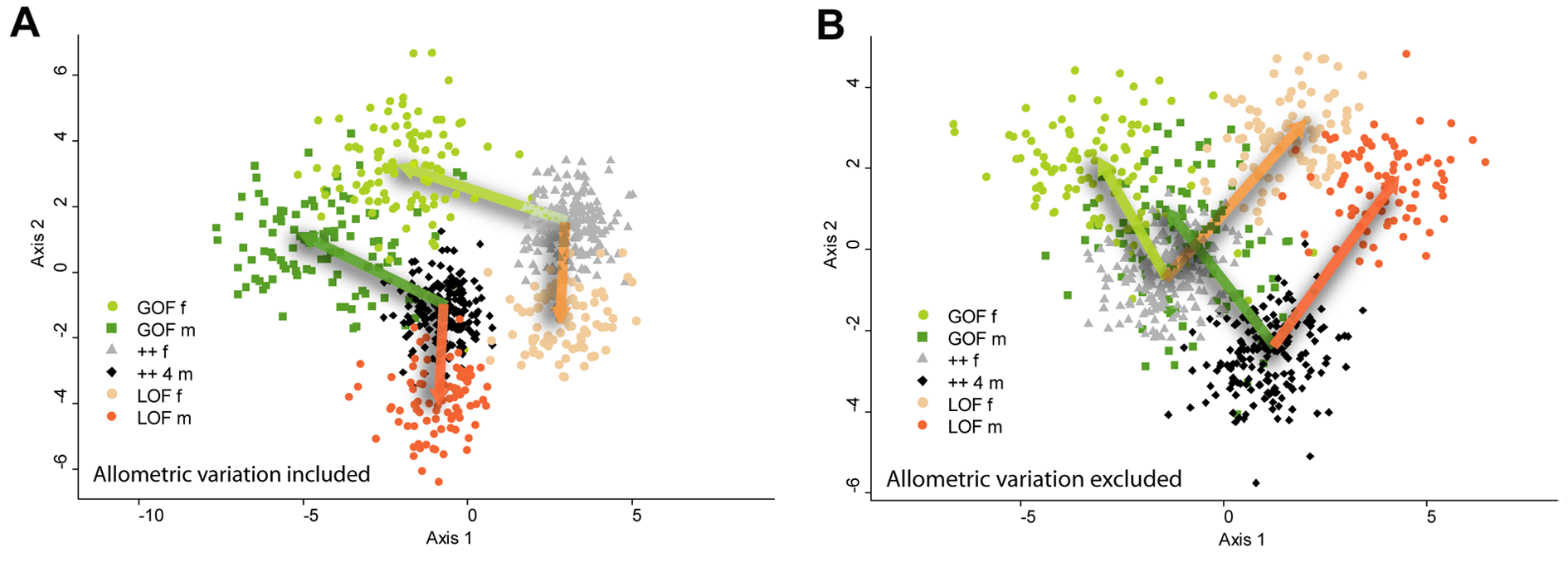

Supplement: Figure S4 — Impact of allometry on mean wing shape discrimination (yw67c2 background). A: Discriminant analysis applied to the shape variables (i.e. non null PC scores; grouping factor: genotype*sex). B: Discriminant analysis applied to the residuals of a multivariate regression of size on shape variables. Grey: +/+ females; black: +/+ males; light green: GOF females; dark green: GOF males; light orange: LOF females; dark orange: LOF males. (TIF) [file pgen.1002314.s004.tif]
