# Supplementary material for: Developmental Stability: A Major Role for Cyclin G in Drosophila melanogaster
Source: PLoS Genet. 2011 Oct 6;7(10):e1002314. doi: 10.1371/journal.pgen.1002314 (PMC3188557; doi:10.1371/journal.pgen.1002314)
Supplement: Table S1 — Effects of CycG deregulation on mean wing size and shape. Mean centroid size values and standard deviations (Sd) are provided. Results of the ANOVAs on centroid size and MANOVAs on the PC scores (genotype and sex as fixed factors). GOF = gain of function; LOF = loss of function; +/+ = controls; f = females; m = males. Sd = standard deviation; Df = degrees of freedom; SS = sum of squares; MS = mean squares; F = Fisher's F value; Pillai = Pillai's Trace; Df den = denominator's degrees of freedom; Df num = numerator's degrees of freedom; * = p<0.05; ** = p<0.01; *** = p<0.001; ns = non significant. (DOC) [file pgen.1002314.s005.doc]

**Table S1: Effects of *CycG* deregulation on mean wing size and shape.**

| **Genetic back-ground** | **Driver** | **Experiment** |  |  |  |  |  |  |  |
| --- | --- | --- | --- | --- | --- | --- | --- | --- | --- |
|  |  |  |  |  |  |  |  |  |  |
| ***w^1118^*** | ***da*** | **GOF** |  |  |  |  |  |  |  |
|  |  |  |  |  |  |  |  |  |  |
|  |  | **GOF f** | **GOF m** | **+/+ f** | **+/+ m** |  |  |  |  |
|  | **Mean size**  **(Sd)** | 0.91  (0.03) | 0.79 (0.04) | 1.07  (0.02) | 0.93  (0.02) |  |  |  |  |
|  |  |  |  |  |  |  |  |  |  |
|  | **Size** | **source of variation** | **Df** | **SS** | **MS** | **F** | **P-value** |  |  |
|  |  | genotype | 1 | 4.53 | 4.53 | 5780.4 | <2.2 x 10^-16^ | *** |  |
|  |  | sex | 1 | 3.26 | 3.26 | 4156.35 | <2.2 x 10^-15^ | *** |  |
|  |  | gen*sex | 1 | 0.02 | 0.02 | 26.09 | 4.07 x 10^-7^ | *** |  |
|  |  | Residuals | 804 | 0.63 | 0.0008 |  |  |  |  |
|  |  |  |  |  |  |  |  |  |  |
|  | **Shape** | **source of variation** | **Df** | **Pillai** | **F** | **Df num** | **Df den** | **P-value** |  |
|  |  | genotype | 1 | 0.87 | 199.47 | 26 | 779 | <2.2 x 10^-16^ | *** |
|  |  | sex | 1 | 0.76 | 95.59 | 26 | 779 | <2.2 x 10^-15^ | *** |
|  |  | gen*sex | 1 | 0.19 | 6.95 | 26 | 779 | <2.2 x 10^-16^ | *** |
|  |  | Residuals | 804 |  |  |  |  |  |  |
|  |  |  |  |  |  |  |  |  |  |
| ***yw67c23*** | ***da*** | **GOF** |  |  |  |  |  |  |  |
|  |  |  |  |  |  |  |  |  |  |
|  |  | **GOF f** | **GOF m** | **+/+ f** | **+/+ m** |  |  |  |  |
|  | **Mean size**  **(Sd)** | 0.86  (0.05) | 0.75  (0.05) | 1.05  (0.2) | 0.91  (0.01) |  |  |  |  |
|  |  |  |  |  |  |  |  |  |  |
|  | **Size** | **source of variation** | **Df** | **SS** | **MS** | **F** | **P-value** |  |  |
|  |  | genotype | 1 | 3.93 | 3.93 | 3969.84 | <2.2 x 10^-16^ | *** |  |
|  |  | sex | 1 | 2.67 | 2.67 | 2699.29 | <2.2 x 10^-15^ | *** |  |
|  |  | gen*sex | 1 | 0.03 | 0.03 | 26.16 | 4.26 x 10^-7^ | *** |  |
|  |  | Residuals | 588 | 0.58 | 0.001 |  |  |  |  |
|  |  |  |  |  |  |  |  |  |  |
|  | **Shape** | **source of variation** | **Df** | **Pillai** | **F** | **Df num** | **Df den** | **P-value** |  |
|  |  | genotype | 1 | 0.86 | 137.66 | 26 | 563 | <2.2 x 10^-16^ | *** |
|  |  | sex | 1 | 0.84 | 113.29 | 26 | 563 | <2.2 x 10^-16^ | *** |
|  |  | gen*sex | 1 | 0.32 | 10.21 | 26 | 563 | <2.2 x 10^-16^ | *** |
|  |  | Residuals | 588 |  |  |  |  |  |  |
|  |  |  |  |  |  |  |  |  |  |
|  |  |  |  |  |  |  |  |  |  |
|  |  |  |  |  |  |  |  |  |  |

**Table S1 (continued):**

| ***yw67c23*** | ***da*** | ***LOF*** |  |  |  |  |  |  |  |
| --- | --- | --- | --- | --- | --- | --- | --- | --- | --- |
|  |  |  |  |  |  |  |  |  |  |
|  |  | **LOF f** | **LOF m** | **+/+ f** | **+/+ m** |  |  |  |  |
|  | **Mean size**  **(Sd)** | 1.04  (0.03) | 0.89 (0.02) | 1.05  (0.01) | 0.89  (0.02) |  |  |  |  |
|  |  |  |  |  |  |  |  |  |  |
|  | **Size** | **source of variation** | **Df** | **SS** | **MS** | **F** | **P-value** |  |  |
|  |  | genotype | 1 | 3.00 x 10^-5^ | 3.00 x 10^-5^ | 0.07 | 0.79 | ns |  |
|  |  | sex | 1 | 2.26 | 2.26 | 5.03 x 10^-3^ | 2.20 x 10^-16^ | *** |  |
|  |  | gen*sex | 1 | 3.16 x 10^-3^ | 3.16 x 10^-3^ | 7.06 | 0.01 | ** |  |
|  |  | Residuals | 370 | 0.17 | 4.50 x 10^-4^ |  |  |  |  |
|  |  |  |  |  |  |  |  |  |  |
|  | **Shape** | **source of variation** | **Df** | **Pillai** | **F** | **Df num** | **Df den** | **P-value** |  |
|  |  | genotype | 1 | 0.9 | 124.39 | 26 | 345 | <2.2 x 10^-16^ | *** |
|  |  | sex | 1 | 0.92 | 142.27 | 26 | 345 | <2.2 x 10^-16^ | *** |
|  |  | gen*sex | 1 | 0.29 | 5.43 | 26 | 345 | 1.93 x 10^-14^ | *** |
|  |  | Residuals | 370 |  |  |  |  |  |  |
|  |  |  |  |  |  |  |  |  |  |
| ***yw67c23*** | ***Act*** | ***GOF*** |  |  |  |  |  |  |  |
|  |  |  |  |  |  |  |  |  |  |
|  |  | **GOF f** | **GOF m** | **+/+ f** | **+/+ m** |  |  |  |  |
|  | **Mean size**  **(Sd)** | 0.88  (0.02) | 0.71 (0.02) | 1.05  (0.01) | 0.90  (0.01) |  |  |  |  |
|  |  |  |  |  |  |  |  |  |  |
|  | **Size** | **source of variation** | **Df** | **SS** | **MS** | **F** | **P-value** |  |  |
|  |  | genotype | 1 | 2.92 | 2.92 | 7971.90 | <2.2 x 10^-16^ | *** |  |
|  |  | sex | 1 | 2.08 | 2.08 | 5686.60 | <2.2 x 10^-16^ | *** |  |
|  |  | gen*sex | 1 | 0.01 | 0.01 | 33.05 | 1.99 x 10^-8^ | *** |  |
|  |  | Residuals | 342 | 0.13 | 0.00037 |  |  |  |  |
|  |  |  |  |  |  |  |  |  |  |
|  | **Shape** | **source of variation** | **Df** | **Pillai** | **F** | **Df num** | **Df den** | **P-value** |  |
|  |  | genotype | 1 | 0.94 | 175.24 | 26 | 317 | <2.2 x 10^-16^ | *** |
|  |  | sex | 1 | 0.88 | 89.87 | 26 | 317 | <2.2 x 10^-16^ | *** |
|  |  | gen*sex | 1 | 0.54 | 14.07 | 26 | 317 | <2.2 x 10^-16^ | *** |
|  |  | Residuals | 342 |  |  |  |  |  |  |
|  |  |  |  |  |  |  |  |  |  |
