# Supplementary material for: Developmental Stability: A Major Role for Cyclin G in Drosophila melanogaster
Source: PLoS Genet. 2011 Oct 6;7(10):e1002314. doi: 10.1371/journal.pgen.1002314 (PMC3188557; doi:10.1371/journal.pgen.1002314)
Supplement: Table S2 — Wing size FA. Results of the two-way mixed model ANOVAs on centroid size (individual = random; side = fixed). The tests are presented for the three control genotypes in the GOF experiments using da as driver (see Material and Methods). FA10 and FA4 are two FA indices (see Material and Methods). FA effect refers to the ratio of the GOF or LOF FA value over the one of the corresponding +/+ genotype. Df = degrees of freedom; MS = mean squares; F = Fisher's F value. MS and FA10 values are multiplied by 105. (DOC) [file pgen.1002314.s006.doc]

Table S2: Wing size FA.

|  |  |  |  |  |  |  |  |  |  |  |  |
| --- | --- | --- | --- | --- | --- | --- | --- | --- | --- | --- | --- |
|  |  |  |  |  |  |  |  |  |  |  |  |
| **Genetic back-ground** | **Driver** | **Experiment** | **Genotype** | **Sex** | **Source of variation** | **Df** | **MS** | **F** | **P-value** | **FA10** | **FA effect** |
|  |  |  |  |  |  |  |  |  |  |  |  |
|  |  |  |  |  |  |  |  |  |  |  |  |
| ***w^1118^*** | ***da*** | ***GOF*** | *+/+* | f | individuals | 45 | 300.7 | 77.1 | < 2.2 x 10^-16^ | 1.912 |  |
|  |  |  |  |  | side | 1 | 12 | 3.08 | 0.09 |  |  |
|  |  |  |  |  | indiv*side | 45 | 3.9 | 38.97 | < 2.2 x 10^-16^ |  |  |
|  |  |  |  |  | residuals | 92 | 0.1 |  |  |  |  |
|  |  |  |  |  |  |  |  |  |  |  |  |
|  |  |  | *+/+* | m | individuals | 47 | 174.8 | 67.23 | < 2.2 x10^-16^ | 1.233 |  |
|  |  |  |  |  | side | 1 | 15.9 | 6.12 | 0.02 |  |  |
|  |  |  |  |  | indiv*side | 47 | 2.6 | 15.3 | < 2.2 x10^-16^ |  |  |
|  |  |  |  |  | residuals | 96 | 0.2 |  |  |  |  |
|  |  |  |  |  |  |  |  |  |  |  |  |
|  |  |  | *da>RCG76* | f | individuals | 54 | 284.6 | 2.02 | 0.005402 | 70.272 | **36.8** |
|  |  |  |  |  | side | 1 | 86.5 | 0.61 | 0.436472 |  |  |
|  |  |  |  |  | indiv*side | 54 | 140.8 | 583.2 | < 2.2 x10^-16^ |  |  |
|  |  |  |  |  | residuals | 110 | 0.2 |  |  |  |  |
|  |  |  |  |  |  |  |  |  |  |  |  |
|  |  |  | *da>RCG76* | m | individuals | 52 | 410.5 | 4.27 | < 2.2 x10^-16^ | 47.917 | **38.9** |
|  |  |  |  |  | side | 1 | 43.5 | 0.45 | 0.504064 |  |  |
|  |  |  |  |  | indiv*side | 52 | 96.1 | 408.9 | < 2.2 x10^-16^ |  |  |
|  |  |  |  |  | residuals | 106 | 0.2 |  |  |  |  |
|  |  |  |  |  |  |  |  |  |  |  |  |
|  |  |  | *da/+* | f | individuals | 47 | 147.3 | 23.76 | < 2.2 x10^-16^ | 2.291 |  |
|  |  |  |  |  | side | 1 | 35.4 | 5.71 | 0.02 |  |  |
|  |  |  |  |  | indiv*side | 47 | 6.2 | 3.87 | < 2.2 x10^-16^ |  |  |
|  |  |  |  |  | residuals | 96 | 1.6 |  |  |  |  |
|  |  |  |  |  |  |  |  |  |  |  |  |
|  |  |  | *da/+* | m | individuals | 47 | 212.1 | 18.28 | < 2.2 x10^-16^ | 4.996 |  |
|  |  |  |  |  | side | 1 | 0.3 | 0.03 | 0.87 |  |  |
|  |  |  |  |  | indiv*side | 47 | 11.6 | 7.03 | < 2.2 x10^-16^ |  |  |
|  |  |  |  |  | residuals | 96 | 1.7 |  |  |  |  |
|  |  |  |  |  |  |  |  |  |  |  |  |
|  |  |  | *RCG76/+* | f | individuals | 46 | 163.7 | 38.07 | < 2.2 x10^-16^ | 2.059 |  |
|  |  |  |  |  | side | 1 | 44.2 | 10.28 | 0.002375 |  |  |
|  |  |  |  |  | indiv*side | 46 | 4.3 | 27.81 | < 2.2 x10^-16^ |  |  |
|  |  |  |  |  | residuals | 94 | 0.2 |  |  |  |  |
|  |  |  |  |  |  |  |  |  |  |  |  |
|  |  |  | *RCG76/+* | m | individuals | 46 | 116.8 | 17.43 | < 2.2 x10^-16^ | 3.255 |  |
|  |  |  |  |  | side | 1 | 0.1 | 0.01 | 0.914997 |  |  |
|  |  |  |  |  | indiv*side | 46 | 6.7 | 32.23 | < 2.2 x10^-16^ |  |  |
|  |  |  |  |  | residuals | 94 | 0.2 |  |  |  |  |
|  |  |  |  |  |  |  |  |  |  |  |  |

**Table S2 (continued)**

| ***yw67c23*** | ***da*** | ***GOF*** | *+/+* | f | individuals | 49 | 146.6 | 35.76 | < 2.2 x10^-16^ | 1.97 |  |
| --- | --- | --- | --- | --- | --- | --- | --- | --- | --- | --- | --- |
|  |  |  |  |  | side | 1 | 4.7 | 1.15 | 0.28498 |  |  |
|  |  |  |  |  | indiv*side | 49 | 4.1 | 32.68 | < 2.2 x10^-16^ |  |  |
|  |  |  |  |  | residuals | 100 | 0.1 |  |  |  |  |
|  |  |  |  |  |  |  |  |  |  |  |  |
|  |  |  | *+/+* | m | individuals | 46 | 68.9 | 19.14 | < 2.2 x10^-16^ | 1.73 |  |
|  |  |  |  |  | side | 1 | 1.2 | 0.33 | 0.569669 |  |  |
|  |  |  |  |  | indiv*side | 46 | 3.6 | 37.39 | < 2.2 x10^-16^ |  |  |
|  |  |  |  |  | residuals | 94 | 0.1 |  |  |  |  |
|  |  |  |  |  |  |  |  |  |  |  |  |
|  |  |  | *da>RCG76* | f | individuals | 24 | 986 | 9.32 | < 2.2 x10^-16^ | 52.85 | **26.9** |
|  |  |  |  |  | side | 1 | 31.4 | 0.3 | 0.59105 |  |  |
|  |  |  |  |  | indiv*side | 24 | 105.8 | 837.7 | < 2.2 x10^-16^ |  |  |
|  |  |  |  |  | residuals | 50 | 0.1 |  |  |  |  |
|  |  |  |  |  |  |  |  |  |  |  |  |
|  |  |  | *da>RCG76* | m | individuals | 25 | 678.7 | 4.06 | < 2.2 x10^-16^ | 83.55 | **48.2** |
|  |  |  |  |  | side | 1 | 5.2 | 0.03 | 0.86085 |  |  |
|  |  |  |  |  | indiv*side | 25 | 167.2 | 1988.6 | < 2.2 x10^-16^ |  |  |
|  |  |  |  |  | residuals | 52 | 0.1 |  |  |  |  |
|  |  |  |  |  |  |  |  |  |  |  |  |
|  |  |  | *da/+* | f | individuals | 48 | 168.1 | 35.02 | < 2.2 x10^-16^ | 2.35 |  |
|  |  |  |  |  | side | 1 | 41.6 | 8.67 | 0.00480 |  |  |
|  |  |  |  |  | indiv*side | 48 | 4.8 | 87.2 | < 2.2 x10^-16^ |  |  |
|  |  |  |  |  | residuals | 98 | 0.1 |  |  |  |  |
|  |  |  |  |  |  |  |  |  |  |  |  |
|  |  |  | *da/+* | m | individuals | 47 | 97.4 | 26.32 | < 2.2 x10^-16^ | 1.83 |  |
|  |  |  |  |  | side | 1 | 28.4 | 7.68 | 0.00818 |  |  |
|  |  |  |  |  | indiv*side | 47 | 3.7 | 57.75 | < 2.2 x10^-16^ |  |  |
|  |  |  |  |  | residuals | 96 | 0.1 |  |  |  |  |
|  |  |  |  |  |  |  |  |  |  |  |  |
|  |  |  | *RCG76/+* | f | individuals | 47 | 181.7 | 75.71 | < 2.2 x10^-16^ | 1.14 |  |
|  |  |  |  |  | side | 1 | 17.5 | 7.29 | 0.00922 |  |  |
|  |  |  |  |  | indiv*side | 47 | 2.4 | 28.04 | < 2.2 x10^-16^ |  |  |
|  |  |  |  |  | residuals | 96 | 0.1 |  |  |  |  |
|  |  |  |  |  |  |  |  |  |  |  |  |
|  |  |  | *RCG76/+* | m | individuals | 49 | 77.2 | 16.08 | < 2.2 x10^-16^ | 2.32 |  |
|  |  |  |  |  | side | 1 | 22.2 | 4.63 | 0.03639 |  |  |
|  |  |  |  |  | indiv*side | 49 | 4.8 | 28.52 | < 2.2 x10^-16^ |  |  |
|  |  |  |  |  | residuals | 100 | 0.2 |  |  |  |  |
|  |  |  |  |  |  |  |  |  |  |  |  |
|  |  |  |  |  |  |  |  |  |  |  |  |
| ***yw67c23*** | ***Act*** | ***GOF*** | *+/+* | f | individuals | 48 | 46.9 | 13.4 | < 2.2 x10^-16^ | 7.01 |  |
|  |  |  |  |  | side | 1 | 0.2 | 0.06 | 0.81014 |  |  |
|  |  |  |  |  | indiv*side | 48 | 3.5 |  |  |  |  |
|  |  |  |  |  |  |  |  |  |  |  |  |
|  |  |  | *+/+* | m | individuals | 49 | 39.1 | 19.76 | < 2.2 x10^-16^ | 3.96 |  |
|  |  |  |  |  | side | 1 | 2.4 | 1.22 | 0.27409 |  |  |
|  |  |  |  |  | indiv*side | 49 | 2.0 |  |  |  |  |

**Table S2 (continued)**

|  |  |  |  |  |  |  |  |  |  |  |  |
| --- | --- | --- | --- | --- | --- | --- | --- | --- | --- | --- | --- |
|  |  |  | *act>RCG76* | f | individuals | 33 | 90.0 | 11.39 | < 2.2 x10^-16^ | 15.81 | **2.26** |
|  |  |  |  |  | side | 1 | 1.1 | 0.14 | 0.70873 |  |  |
|  |  |  |  |  | indiv*side | 33 | 7.9 |  |  |  |  |
|  |  |  |  |  |  |  |  |  |  |  |  |
|  |  |  | *act>RCG76* | m | individuals | 39 | 119.4 | 24.88 | < 2.2 x10^-16^ | 9.61 | **2.43** |
|  |  |  |  |  | side | 1 | 14.5 | 3.02 | 0.09053 |  |  |
|  |  |  |  |  | indiv*side | 39 | 4.8 |  |  |  |  |
|  |  |  |  |  |  |  |  |  |  |  |  |
|  |  |  |  |  |  |  |  |  |  |  |  |
| ***yw67c23*** | ***sd*** | ***GOF*** | *+/+* | f | individuals | 48 | 89 | 15.42 | < 2.2 x10^-16^ | 11.58 |  |
|  |  |  |  |  | side | 1 | 2 | 0.28 | 0.6 |  |  |
|  |  |  |  |  | indiv*side | 48 | 6 |  |  |  |  |
|  |  |  |  |  |  |  |  |  |  |  |  |
|  |  |  | *+/+* | m | individuals | 49 | 72 | 29.28 | < 2.2 x10^-16^ | 4.89 |  |
|  |  |  |  |  | side | 1 | 22 | 8.81 | 4.62 x10^-3^ |  |  |
|  |  |  |  |  | indiv*side | 49 | 2 |  |  |  |  |
|  |  |  |  |  |  |  |  |  |  |  |  |
|  |  |  | *sd>RCG76* | f | individuals | 44 | 497 | 12.69 | < 2.2 x10^-16^ | 78.32 | **6.76** |
|  |  |  |  |  | side | 1 | 71 | 1.82 | 0.18 |  |  |
|  |  |  |  |  | indiv*side | 44 | 39 |  |  |  |  |
|  |  |  |  |  |  |  |  |  |  |  |  |
|  |  |  | *sd>RCG76* | m | individuals | 49 | 1451 | 12.39 | < 2.2 x10^-16^ | 234.23 | **47.93** |
|  |  |  |  |  | side | 1 | 17 | 0.14 | 0.71 |  |  |
|  |  |  |  |  | indiv*side | 49 | 117 |  |  |  |  |
|  |  |  |  |  |  |  |  |  |  |  |  |
|  |  |  |  |  |  |  |  |  |  |  |  |
| ***yw67c23*** | ***da*** | ***LOF*** | *+/+* | f | individuals | 49 | 73.75 | 35.29 | < 2.2 x10^-16^ | 4.19 |  |
|  |  |  |  |  | side | 1 | 2.2 | 1.05 | 0.69 |  |  |
|  |  |  |  |  | indiv*side | 49 | 2.09 |  |  |  |  |
|  |  |  |  |  |  |  |  |  |  |  |  |
|  |  |  | *+/+* | m | individuals | 46 | 34.32 | 19.5 | < 2.2 x10^-16^ | 3.53 |  |
|  |  |  |  |  | side | 1 | 0.68 | 0.39 | 0.46 |  |  |
|  |  |  |  |  | indiv*side | 46 | 1.76 |  |  |  |  |
|  |  |  |  |  |  |  |  |  |  |  |  |
|  |  |  | *da>dscycG2* | f | individuals | 44 | 180.1 | 85.83 | < 2.2 x10^-16^ | 4.2 | **1.00** |
|  |  |  |  |  | side | 1 | 0.2 | 0.07 | 0.79 |  |  |
|  |  |  |  |  | indiv*side | 44 | 2.1 |  |  |  |  |
|  |  |  |  |  |  |  |  |  |  |  |  |
|  |  |  | *da>dscycG2* | m | individuals | 43 | 81.1 | 29.86 | < 2.2 x10^-16^ | 5.43 | **1.54** |
|  |  |  |  |  | side | 1 | 5.6 | 2.06 | 0.16 |  |  |
|  |  |  |  |  | indiv*side | 43 | 2.7 |  |  |  |  |
