# Supplementary material for: Developmental Stability: A Major Role for Cyclin G in Drosophila melanogaster
Source: PLoS Genet. 2011 Oct 6;7(10):e1002314. doi: 10.1371/journal.pgen.1002314 (PMC3188557; doi:10.1371/journal.pgen.1002314)
Supplement: Table S3 — Wing shape FA. Results of the Procrustes ANOVAs (individual and side as main effects). MS and FA10 values are multiplied by 107. Df = degrees of freedom; MS = mean squares; F = Fisher's F value. (DOC) [file pgen.1002314.s007.doc]

**Table S3: Wing shape FA.**

| **Genetic back-ground** | **Driver** | **Experiment** | **Genotype** | **Sex** | **Source of variation** | **Df** | **MS** | **F** | **P-value** | **FA10** | **FA effect** |
| --- | --- | --- | --- | --- | --- | --- | --- | --- | --- | --- | --- |
|  |  |  |  |  |  |  |  |  |  |  |  |
| ***w^1118^*** | ***da*** | ***GOF*** | *+/+* | f | individuals | 1170 | 51.67 | 8.07 | < 2.2 x10^-16^ | 5.86 |  |
|  |  |  |  |  | side | 26 | 22.84 | 3.57 | 5.23 x10^-9^ |  |  |
|  |  |  |  |  | indiv*side | 1170 | 6.4 | 11.84 | < 2.2 x10^-16^ |  |  |
|  |  |  |  |  | residuals | 2392 | 0.54 |  |  |  |  |
|  |  |  |  |  |  |  |  |  |  |  |  |
|  |  |  | *+/+* | m | individuals | 1222 | 44.93 | 7.27 | < 2.2 x10^-16^ | 5.6 |  |
|  |  |  |  |  | side | 26 | 29.73 | 4.81 | 4.41 X10^-14^ |  |  |
|  |  |  |  |  | indiv*side | 1222 | 6.18 | 10.77 | < 2.2 x10^-16^ |  |  |
|  |  |  |  |  | residuals | 2496 | 0.57 |  |  |  |  |
|  |  |  |  |  |  |  |  |  |  |  |  |
|  |  |  | *da>RCG76* | f | individuals | 1404 | 121.15 | 2.61 | < 2.2 x10^-16^ | 44.51 | **7.59** |
|  |  |  |  |  | side | 26 | 2.67 | 0.06 | 1 |  |  |
|  |  |  |  |  | indiv*side | 1404 | 46.36 | 24.96 | 0 |  |  |
|  |  |  |  |  | residuals | 2860 | 1.86 |  |  |  |  |
|  |  |  |  |  |  |  |  |  |  |  |  |
|  |  |  | *da>RCG76* | m | individuals | 1352 | 98.29 | 1.66 | 0 | 57.02 | **10.18** |
|  |  |  |  |  | side | 26 | 187.91 | 3.17 | 1.69 x10^-7^ |  |  |
|  |  |  |  |  | indiv*side | 1352 | 59.36 | 25.32 | < 2.2 x10^-16^ |  |  |
|  |  |  |  |  | residuals | 2756 | 2.34 |  |  |  |  |
|  |  |  |  |  |  |  |  |  |  |  |  |
|  |  |  | *da/+* | f | individuals | 1222 | 60.89 | 6.88 | < 2.2 x10^-16^ | 8.22 |  |
|  |  |  |  |  | side | 26 | 3.84 | 0.43 | 0.99 |  |  |
|  |  |  |  |  | indiv*side | 1222 | 8.85 | 14.12 | < 2.2 x10^-16^ |  |  |
|  |  |  |  |  | residuals | 2496 | 0.63 |  |  |  |  |
|  |  |  |  |  |  |  |  |  |  |  |  |
|  |  |  | *da/+* | m | individuals | 1222 | 34.02 | 7.63 | < 2.2 x10^-16^ | 3.75 |  |
|  |  |  |  |  | side | 26 | 25.98 | 5.83 | < 2.2 x10^-16^ |  |  |
|  |  |  |  |  | indiv*side | 1222 | 4.46 | 6.34 | < 2.2 x10^-16^ |  |  |
|  |  |  |  |  | residuals | 2496 | 0.7 |  |  |  |  |
|  |  |  |  |  |  |  |  |  |  |  |  |
|  |  |  | *RCG76/+* | f | individuals | 1196 | 47.01 | 8.63 | < 2.2 x10^-16^ | 3.79 |  |
|  |  |  |  |  | side | 26 | 32.77 | 6.01 | < 2.2 x10^-16^ |  |  |
|  |  |  |  |  | indiv*side | 1196 | 5.45 | 3.29 | < 2.2 x10^-16^ |  |  |
|  |  |  |  |  | residuals | 2444 | 1.65 |  |  |  |  |
|  |  |  |  |  |  |  |  |  |  |  |  |
|  |  |  | *RCG76/+* | m | individuals | 1196 | 40.37 | 4.42 | < 2.2 x10^-16^ | 7.6 |  |
|  |  |  |  |  | side | 26 | 7.01 | 0.77 | 0.79 |  |  |
|  |  |  |  |  | indiv*side | 1196 | 9.14 | 5.93 | < 2.2 x10^-16^ |  |  |
|  |  |  |  |  | residuals | 2444 | 1.54 |  |  |  |  |
|  |  |  |  |  |  |  |  |  |  |  |  |
| ***yw67c23*** | ***da*** | ***GOF*** | *+/+* | f | individuals | 1274 | 22.93 | 4.39 | < 2.2 x10^-16^ | 5.14 |  |
|  |  |  |  |  | side | 26 | 20.71 | 3.97 | 1.24 x10^-10^ |  |  |
|  |  |  |  |  | indiv*side | 1274 | 5.22 | 68.51 | < 2.2 x10^-16^ |  |  |
|  |  |  |  |  | residuals | 2600 | 0.08 |  |  |  |  |
|  |  |  |  |  |  |  |  |  |  |  |  |

Table S3 (continued)

|  |  |  | *+/+* | m | individuals | 1196 | 13.78 | 2.23 | < 2.2 x10^-16^ | 6.08 |  |
| --- | --- | --- | --- | --- | --- | --- | --- | --- | --- | --- | --- |
|  |  |  |  |  | side | 26 | 31.42 | 5.09 | 3.22 x10^-15^ |  |  |
|  |  |  |  |  | indiv*side | 1196 | 6.17 | 68.29 | < 2.2 x10^-16^ |  |  |
|  |  |  |  |  | residuals | 2444 | 0.09 |  |  |  |  |
|  |  |  |  |  |  |  |  |  |  |  |  |
|  |  |  | *da>RCG76* | f | individuals | 624 | 185.92 | 3.91 | < 2.2 x10^-16^ | 47.3 | **9.19** |
|  |  |  |  |  | side | 26 | 164.05 | 3.45 | 3.11 x10^-8^ |  |  |
|  |  |  |  |  | indiv*side | 624 | 47.6 | 155.65 | < 2.2 x10^-16^ |  |  |
|  |  |  |  |  | residuals | 1300 | 0.31 |  |  |  |  |
|  |  |  |  |  |  |  |  |  |  |  |  |
|  |  |  | *da>RCG76* | m | individuals | 650 | 104.83 | 1.54 | 1.87 X10^-8^ | 67.75 | **11.14** |
|  |  |  |  |  | side | 26 | 0.01 | 0.0002 | 1 |  |  |
|  |  |  |  |  | indiv*side | 650 | 67.96 | 333.96 | < 2.2 x10^-16^ |  |  |
|  |  |  |  |  | residuals | 1352 | 0.2 |  |  |  |  |
|  |  |  |  |  |  |  |  |  |  |  |  |
|  |  |  | *da/+* | f | individuals | 1248 | 35.92 | 2.83 | < 2.2 x10^-16^ | 12.54 |  |
|  |  |  |  |  | side | 26 | 0.72 | 0.06 | 1 |  |  |
|  |  |  |  |  | indiv*side | 1248 | 12.69 | 84.62 | < 2.2 x10^-16^ |  |  |
|  |  |  |  |  | residuals | 2548 | 0.15 |  |  |  |  |
|  |  |  |  |  |  |  |  |  |  |  |  |
|  |  |  | *da/+* | m | individuals | 1222 | 23.7 | 2.21 | < 2.2 x10^-16^ | 10.59 |  |
|  |  |  |  |  | side | 26 | 11.91 | 1.11 | 0.322 |  |  |
|  |  |  |  |  | indiv*side | 1222 | 10.75 | 70.63 | < 2.2 x10^-16^ |  |  |
|  |  |  |  |  | residuals | 2496 | 0.15 |  |  |  |  |
|  |  |  |  |  |  |  |  |  |  |  |  |
|  |  |  | *RCG76/+* | f | individuals | 1222 | 48.46 | 8.8 | < 2.2 x10^-16^ | 5.37 |  |
|  |  |  |  |  | side | 26 | 9.2 | 1.67 | 0.019 |  |  |
|  |  |  |  |  | indiv*side | 1222 | 5.5 | 42.8 | < 2.2 x10^-16^ |  |  |
|  |  |  |  |  | residuals | 2496 | 0.13 |  |  |  |  |
|  |  |  |  |  |  |  |  |  |  |  |  |
|  |  |  | *RCG76/+* | m | individuals | 1274 | 16.62 | 3.15 | < 2.2 x10^-16^ | 5.1 |  |
|  |  |  |  |  | side | 26 | 4.56 | 0.86 | 0.661 |  |  |
|  |  |  |  |  | indiv*side | 1274 | 5.27 | 30.45 | < 2.2 x10^-16^ |  |  |
|  |  |  |  |  | residuals | 2600 | 0.17 |  |  |  |  |
|  |  |  |  |  |  |  |  |  |  |  |  |
| ***yw67c23*** | ***Act*** | ***GOF*** | *+/+* | f | individuals | 1248 | 14.96 | 4.69 | < 2.2 x10^-16^ | 3.19 |  |
|  |  |  |  |  | side | 26 | 3.15 | 0.99 | 0.48 |  |  |
|  |  |  |  |  | indiv*side | 1248 | 3.19 |  |  |  |  |
|  |  |  |  |  |  |  |  |  |  |  |  |
|  |  |  | *+/+* | m | individuals | 1274 | 20.31 | 7.7 | < 2.2 x10^-16^ | 2.64 |  |
|  |  |  |  |  | side | 26 | 1.59 | 0.6 | 0.94 |  |  |
|  |  |  |  |  | indiv*side | 1274 | 2.64 |  |  |  |  |
|  |  |  |  |  |  |  |  |  |  |  |  |
|  |  |  | *act>RCG76* | f | individuals | 858 | 72.03 | 2.3 | < 2.2 x10^-16^ | 31.37 | **9.83** |
|  |  |  |  |  | side | 26 | 42.24 | 1.35 | 0.12 |  |  |
|  |  |  |  |  | indiv*side | 858 | 31.37 |  |  |  |  |
|  |  |  |  |  |  |  |  |  |  |  |  |
|  |  |  | *act>RCG76* | m | individuals | 1014 | 52.31 | 7.09 | < 2.2 x10^-16^ | 7.38 | **2.80** |
|  |  |  |  |  | side | 26 | 9.27 | 1.26 | 0.18 |  |  |
|  |  |  |  |  | indiv*side | 1014 | 7.38 |  |  |  |  |

Table S3 (continued)

| ***yw67c23*** | ***da*** | ***LOF*** | *+/+* | f | individuals | 1222 | 12.89 | 4.67 | < 2.2 x10^-16^ | 2.76 |  |
| --- | --- | --- | --- | --- | --- | --- | --- | --- | --- | --- | --- |
|  |  |  |  |  | side | 26 | 2.02 | 0.73 | 0.83 |  |  |
|  |  |  |  |  | indiv*side | 1222 | 2.76 |  |  |  |  |
|  |  |  |  |  |  |  | 0 |  |  |  |  |
|  |  |  | *+/+* | m | individuals | 1274 | 12.89 | 3.41 | < 2.2 x10^-16^ | 3.78 |  |
|  |  |  |  |  | side | 26 | 7.21 | 1.91 | 3.98 X10^-3^ |  |  |
|  |  |  |  |  | indiv*side | 1274 | 3.78 |  |  |  |  |
|  |  |  |  |  |  |  |  |  |  |  |  |
|  |  |  | *da>dscycG2* | f | individuals | 1144 | 22.4 | 2.19 | < 2.2 x10^-16^ | 10.22 | **3.71** |
|  |  |  |  |  | side | 26 | 1.05 | 0.1 | 1 |  |  |
|  |  |  |  |  | indiv*side | 1144 | 10.22 |  |  |  |  |
|  |  |  |  |  |  |  | 0 |  |  |  |  |
|  |  |  | *da>dscycG2* | m | individuals | 1118 | 19.89 | 2.26 | < 2.2 x10^-16^ | 8.79 | **2.33** |
|  |  |  |  |  | side | 26 | 3.2 | 0.36 | 0.99 |  |  |
|  |  |  |  |  | indiv*side | 1118 | 8.79 |  |  |  |  |
|  |  |  |  |  |  |  |  |  |  |  |  |
|  |  |  |  |  |  |  |  |  |  |  |  |
