# Supplementary material for: Developmental Stability: A Major Role for Cyclin G in Drosophila melanogaster
Source: PLoS Genet. 2011 Oct 6;7(10):e1002314. doi: 10.1371/journal.pgen.1002314 (PMC3188557; doi:10.1371/journal.pgen.1002314)
Supplement: Table S4 — Comparison of patterns of shape FA (females). Correlation of PC1s of FA matrices for all genotypes as measured by the angles among PCs. In brackets are the associated P-values. Top: H0 = the null hypothesis is that the angles between FA PCs are not different from those between pairs of random vectors (10000 random 26 dimensional vectors). A significant effect means that the correlation is stronger than expected from chance only. The P-value is computed as [1 -(number of random angles larger than the observed one)]/10000. Bottom: H0 = the null hypothesis is that the angles between FA PCs are not larger from those between pairs of vectors differing only by the sampling error. Statistical significance is tested against a null distribution of vectors derived from a within genotype boostrap procedure (x10000). A non significant effect means that the vectors are as strongly correlated as vectors differing only by sampling error (i.e. they are almost identical). The P-value is computed as [(number of bootstrapped angles larger than the observed one)]/10000. * = p <0.05; ** = p<0.01; *** = p<0.001, ns = non significant. (DOC) [file pgen.1002314.s008.doc]

**Table S4: Comparison of patterns of shape FA (females).**

| **Angle (P-value)** | |  | ***w^1118^*** | | ***yw^67c23^*** | | |
| --- | --- | --- | --- | --- | --- | --- | --- |
|  |  |  |  |  |  |  |  |
| H0: random variation | |  | ***GOF*** | ***+/+*** | ***GOF*** | ***+/+*** | ***LOF*** |
|  |  |  |  |  |  |  |  |
|  | ***w^1118^*** | ***GOF*** | - |  |  |  |  |
|  |  | **+/+** | 37.7  (0***) | - |  |  |  |
|  |  |  |  |  |  |  |  |
|  | ***yw^67c23^*** | ***GOF*** | 14.47  (0 ***) | 41.7  (0 ***) | - |  |  |
|  |  | **+/+** | 63.37  (0.014 *) | 70.85  (0.06 ns) | 65.56  (0.02*) | - |  |
|  |  | ***LOF*** | 31.7  (0 ***) | 57.26  (0.0014 ***) | 35.27  (0***) | 57.42  (0***) | - |
|  |  |  |  |  |  |  |  |
| H0: sampling variation | |  | ***GOF*** | ***+/+*** | ***GOF*** | ***+/+*** | ***LOF*** |
|  |  |  |  |  |  |  |  |
|  | ***w^1118^*** | ***GOF*** | - |  |  |  |  |
|  |  | ***+/+*** | 37.7  (0.49 ns) | - |  |  |  |
|  |  |  |  |  |  |  |  |
|  | ***yw^67c23^*** | ***GOF*** | 14.47  (0.25 ns) | 41.7  (0.5 ns) | - |  |  |
|  |  | ***+/+*** | 63.37  (0***) | 70.85  (0.22 ns) | 65.56  (0.01*) | - |  |
|  |  | ***LOF*** | 31.7  (0.09 ns) | 57.26  (0***) | 35.27  (0.1 ns) | 57.4  (0 ***) | - |
