# Supplementary material for: Developmental Stability: A Major Role for Cyclin G in Drosophila melanogaster
Source: PLoS Genet. 2011 Oct 6;7(10):e1002314. doi: 10.1371/journal.pgen.1002314 (PMC3188557; doi:10.1371/journal.pgen.1002314)
Supplement: Table S5 — Femur length FA. Results of the two-way mixed model ANOVAs on femur length (individual = random; side = fixed). Df = degrees of freedom; MS = mean squares; F = Fisher's F value. (DOC) [file pgen.1002314.s009.doc]

Table S5: Femur length FA.

| **Genetic background** | **Genotype** | **Sex** | **Source of variation** | **Df** | **MS** | **F** | **P-value** | **FA10** | **FA effect** |
| --- | --- | --- | --- | --- | --- | --- | --- | --- | --- |
|  |  |  |  |  |  |  |  |  |  |
| ***yw67c23*** | *+/+* | f | individuals | 46 | 286.8 | 8.45 | 1.46 x 10^-11^ | 10.51 |  |
|  |  |  | side | 1 | 11.2 | 0.33 | 0.57 |  |  |
|  |  |  | indiv*side | 46 | 34 | 13.93 | < 2.2 x 10^-16^ |  |  |
|  |  |  | residuals | 188 | 2.4 |  |  |  |  |
|  |  |  |  |  |  |  |  |  |  |
|  | *+/+* | m | individuals | 45 | 252.3 | 4.32 | 1.36 x10^-6^ | 18.75 |  |
|  |  |  | side | 1 | 13.5 | 0.23 | 0.63 |  |  |
|  |  |  | indiv*side | 45 | 58.3 | 28.2 | < 2.2 x 10^-16^ |  |  |
|  |  |  | residuals | 184 | 2.1 |  |  |  |  |
|  |  |  |  |  |  |  |  |  |  |
|  | *da>RCG76* | f | individuals | 48 | 1030 | 7.58 | 4.19 x 10^-11^ | 44.36 | **4.22** |
|  |  |  | side | 1 | 601 | 4.42 | 0.04 |  |  |
|  |  |  | indiv*side | 48 | 136 | 7.46 | < 2.2 x 10^-16^ |  |  |
|  |  |  | residuals | 196 | 3 |  |  |  |  |
|  |  |  |  |  |  |  |  |  |  |
|  | *da>RCG76* | m | individuals | 32 | 1364 | 9.22 | 4.97 x 10^-9^ | 47.98 | **2.56** |
|  |  |  | side | 1 | 1 | 0.01 | 0.93 |  |  |
|  |  |  | indiv*side | 32 | 148 | 7.57 | < 2.2 x 10^-16^ |  |  |
|  |  |  | residuals | 132 | 4 |  |  |  |  |
|  |  |  |  |  |  |  |  |  |  |
|  | *da/+* | f | individuals | 46 | 190.8 | 4.37 | 9.15 x 10^-7^ | 13.61 |  |
|  |  |  | side | 1 | 58.6 | 1.34 | 0.25 |  |  |
|  |  |  | indiv*side | 46 | 43.7 | 15.17 | < 2.2 x 10^-16^ |  |  |
|  |  |  | residuals | 188 | 2.9 |  |  |  |  |
|  |  |  |  |  |  |  |  |  |  |
|  | *da/+* | m | individuals | 47 | 234.6 | 6.25 | 2.05 x 10^-9^ | 11.69 |  |
|  |  |  | side | 1 | 2.2 | 0.06 | 0.81 |  |  |
|  |  |  | indiv*side | 47 | 37.5 | 15.11 | < 2.2 x 10^-16^ |  |  |
|  |  |  | residuals | 192 | 2.5 |  |  |  |  |
|  |  |  |  |  |  |  |  |  |  |
|  | *RCG76/+* | f | individuals | 46 | 198 | 5.85 | 9.19 x 10^-9^ | 10.67 |  |
|  |  |  | side | 1 | 3.39 x 10^-3^ | 1.00 x 10^-4^ | 0.99 |  |  |
|  |  |  | indiv*side | 46 | 33 | 18.15 | < 2.2 x 10^-16^ |  |  |
|  |  |  | residuals | 188 | 1 | 9 |  |  |  |
|  |  |  |  |  |  |  |  |  |  |
|  | *RCG76/+* | m | individuals | 47 | 298.9 | 4.89 | 1.21 x 10^-7^ | 19.72 |  |
|  |  |  | side | 1 | 29 | 0.47 | 0.49 |  |  |
|  |  |  | indiv*side | 47 | 61.1 | 31.39 | < 2.2 x 10^-16^ |  |  |
|  |  |  | residuals | 192 | 1.9 |  |  |  |  |
|  |  |  |  |  |  |  |  |  |  |
