# Supplementary material for: Developmental Stability: A Major Role for Cyclin G in Drosophila melanogaster
Source: PLoS Genet. 2011 Oct 6;7(10):e1002314. doi: 10.1371/journal.pgen.1002314 (PMC3188557; doi:10.1371/journal.pgen.1002314)
Supplement: Table S6 — Effects on mean cell size and cell number. Mean wing cell size and cell number and standard deviations (Sd) are provided. Note that cell size is in arbitrary unit, being computed as one divided by the number of cells counted in the standardized area (see Figure 3C′). Cell number strongly underestimate the real cell number in the wing: only one side of the wing was considered (dorsal); the wing basis was not included in the analysis; cells on - or at the direct vicinity of - veins were excluded due to the image thresholding (see Figure 3C″. This underestimation is likely similar across individuals and genotypes and should thus not affect the results. ANOVAs (genotype and sex as main fixed effects) are presented with post hoc Tukey HSD test. Df = degrees of freedom; SS = sums of squares; MS = mean squares; F = Fisher's F-test value; Diff = Difference in the observed means; lwr and upr = lower and upper limits of the interval range for each comparison; p-adj = adjusted P-value; * = p <0.05; *** = p<0.001, ns = non significant. (DOC) [file pgen.1002314.s010.doc]

Table S6: Effects on mean cell size and cell number.

| **Cell size (CS)** | | |  |  |  |  |  |  |
| --- | --- | --- | --- | --- | --- | --- | --- | --- |
|  | ***+/+ f*** | | ***+/+ m*** | ***GOF f*** | ***GOF m*** | ***LOF f*** | ***LOF m*** |  |
| **mean value** | 0.0024 | | 0.0024 | 0.0021 | 0.0019 | 0.0031 | 0.0026 |  |
| **Sd** | 0.000123 | | 0.000109 | 0.000137 | 0.000136 | 0.000296 | 0.000189 |  |
|  |  | |  |  |  |  |  |  |
| **ANOVA** | **source of variation** | | **Df** | **SS** | **MS** | **F** | **P-value** |  |
|  | genotype | | 2 | 2.8 | 1.4 | 287.3 | <2.2 x 10^-16^ | *** |
|  | sex | | 1 | 0.23 | 0.23 | 46.41 | 2.52 x 10^-10^ | *** |
|  | gen*sex | | 2 | 0.17 | 0.08 | 17.31 | 1.88 x 10^-7^ | *** |
|  | Residuals | | 142 | 0.69 | 0 |  |  |  |
|  |  | |  |  |  |  |  |  |
| **Tukey HSD** |  | | **diff** | **lwr** | **upr** | **p-adj** |  |  |
|  | *GOF f - +/+ f* | | -0.13 | -0.19 | -0.07 | 1.00 X10^-7^ | *** |  |
|  | *LOF f - +/+ f* | | 0.25 | 0.19 | 0.3 | <2.2 X10^-16^ | *** |  |
|  | *+/+ m - +/+ f* | | 0.02 | -0.05 | 0.08 | 9.80 X10^-1^ | *** |  |
|  | *GOF m - +/+ f* | | -0.21 | -0.27 | -0.15 | <2.2 X10^-16^ | *** |  |
|  | *LOF m - +/+ f* | | 0.1 | 0.04 | 0.15 | 1.05 X10^-5^ | *** |  |
|  | *LOF f - GOF f* | | 0.38 | 0.32 | 0.43 | <2.2 X10^-16^ | *** |  |
|  | *+/+ m - GOF f* | | 0.14 | 0.08 | 0.21 | <2.2 X10^-16^ | *** |  |
|  | *GOF m - GOF f* | | -0.08 | -0.14 | -0.02 | 4.60 X10^-3^ | *** |  |
|  | *LOF m - GOF f* | | 0.23 | 0.17 | 0.28 | <2.2 X10^-16^ | *** |  |
|  | *+/+ m - LOF f* | | -0.23 | -0.29 | -0.17 | <2.2 X10^-16^ | *** |  |
|  | *GOF m - LOF f* | | -0.45 | -0.51 | -0.4 | <2.2 X10^-16^ | *** |  |
|  | *LOF m - LOF f* | | -0.15 | -0.2 | -0.1 | <2.2 X10^-16^ | *** |  |
|  | *GOF m - +/+ m* | | -0.22 | -0.28 | -0.16 | <2.2 X10^-16^ | *** |  |
|  | *LOF m - +/+ m* | | 0.08 | 0.03 | 0.14 | 4.94 X10^-4^ | *** |  |
|  | *LOF m - GOF m* | | 0.31 | 0.25 | 0.36 | <2.2 X10^-16^ | *** |  |
|  |  | |  |  |  |  |  |  |
| **Cell number (N)** | | |  |  |  |  |  |  |
|  | | ***+/+ f*** | ***+/+ m*** | ***GOF f*** | ***GOF m*** | ***LOF f*** | ***LOF m*** |  |
| **mean value** | | 16270.96 | 12531.98 | 14099.98 | 11797.12 | 12514.74 | 11701.18 |  |
| **Sd** | | 998.14 | 914.88 | 1114.09 | 818.36 | 1528.21 | 770.75 |  |
|  | |  |  |  |  |  |  |  |
| **ANOVA** | | **source of variation** | **Df** | **SS** | **MS** | **F** | **P-value** |  |
|  | | genotype | 2 | 0.8 | 0.4 | 58.47 | <2.2 X10^-16^ | *** |
|  | | sex | 1 | 0.9 | 0.9 | 131.64 | <2.2 X10^-16^ | *** |
|  | | gen*sex | 2 | 0.26 | 0.13 | 19.04 | 4.74 X10^-8^ | *** |
|  | | Residuals | 142 | 0.97 | 0.01 |  |  |  |
|  | |  |  |  |  |  |  |  |
|  | |  |  |  |  |  |  |  |
| **Tukey HSD** | |  | **diff** | **lwr** | **upr** | **p-adj** |  |  |
|  | | *GOF f - +/+ f* | -0.14 | -0.21 | -0.07 | 3.00 X10^-7^ | *** |  |
|  | | *LOF f - +/+ f* | -0.27 | -0.34 | -0.2 | <2.2 X10^-16^ | *** |  |
|  | | *+/+ m - +/+ f* | -0.26 | -0.33 | -0.19 | <2.2 X10^-16^ | *** |  |
|  | | *GOF m - +/+ f* | -0.32 | -0.39 | -0.25 | <2.2 X10^-16^ | *** |  |
|  | | *LOF m - +/+ f* | -0.33 | -0.39 | -0.27 | <2.2 X10^-16^ | *** |  |
|  | | *LOF f - GOF f* | -0.12 | -0.19 | -0.06 | 7.00 X10^-6^ | *** |  |
|  | | *+/+ m - GOF f* | -0.12 | -0.19 | -0.05 | 8.25 X10^-5^ | *** |  |
|  | | *GOF m - GOF f* | -0.18 | -0.25 | -0.11 | <2.2 X10^-16^ | *** |  |
|  | | *LOF m - GOF f* | -0.19 | -0.25 | -0.12 | <2.2 X10^-16^ | *** |  |
|  | | *+/+ m - LOF f* | 0.01 | -0.06 | 0.08 | 0.9998 | ns |  |
|  | | *GOF m - LOF f* | -0.05 | -0.12 | 0.02 | 0.2217 | ns |  |
|  | | *LOF m - LOF f* | -0.06 | -0.12 | 0 | 0.0495 | ns |  |
|  | | *GOF m - +/+ m* | -0.06 | -0.13 | 0.01 | 0.1757 | ns |  |
|  | | *LOF m - +/+ m* | -0.07 | -0.13 | 0 | 0.0413 | * |  |
|  | | *LOF m - GOF m* | -0.01 | -0.07 | 0.06 | 0.9994 | ns |  |
