# Supplementary material for: Developmental Stability: A Major Role for Cyclin G in Drosophila melanogaster
Source: PLoS Genet. 2011 Oct 6;7(10):e1002314. doi: 10.1371/journal.pgen.1002314 (PMC3188557; doi:10.1371/journal.pgen.1002314)
Supplement: Table S7 — Covariation between cell size and cell number. Top: Pearson 's correlation coefficients (r) were computed for each genotypes. They were then compared statistically after applying a Fisher r to Z transformation. Bottom: Analysis of the residuals of regressions of cell size over cell number for each genotype and sex. Statistical significance of variance differences are tested using standard F tests. Z = Fisher Z values; F = Fisher F; p = P-values. (DOC) [file pgen.1002314.s011.doc]

Table S7: Covariation between cell size and cell number

|  | ***GOF m*** | ***GOF f*** | ***+/+ m*** | ***+/+ f*** | ***LOF m*** | ***LOF f*** |
| --- | --- | --- | --- | --- | --- | --- |
| **Pearson's r** | 0.037  p = 0.87  ns | -0.51  p = 0.01  * | -0.83  p = 2.16 x10^-6^ *** | -0.77  p = 1.29 x10^-5^ *** | -0.76  p = 1.95 x10^-7^ *** | -0.91  p = 5.71 x10^-11^ *** |
|  |  |  |  |  |  |  |
| ***GOF m*** | - |  |  |  |  |  |
| ***GOF f*** | Z = 3.645  p = 2.26x10^-4^ | - |  |  |  |  |
| ***+/+ m*** | Z = 7.295  p = 2.97x10^-13^ | Z = 3.849  p =1.18x10^-4^ | - |  |  |  |
| ***+/+ f*** | Z = 6.447  p =1.14x10^-11^ | Z = 2.882  p = 0.0039 | Z = 1.048  p = 0.2940 | - |  |  |
| ***LOF m*** | Z = 6.878  p = 6.06x10^-12^ | Z = 2.994  p = 0.0027 | Z = 1.313  p = 0.1880 | Z = 0.173  p = 0.8610 | - |  |
| ***LOF f*** | Z = 9.738  p = 0 | Z = 6.121  p = 9.25x10^-10^ | Z = 1.915  p = 0.0550 | Z = 3.104  p =0.0019 | Z = 3.631  p = 2.81x10^-4^ | - |
|  |  |  |  |  |  |  |
|  | ***GOF m*** | ***GOF f*** | ***+/+ m*** | ***+/+ f*** | ***LOF m*** | ***LOF f*** |
| **variance of residuals** | 1.84 x 10^-8^ | 1.37 x 10^-8^ | 3.53 x 10^-9^ | 6.03 x 10^-9^ | 1.47 x 10^-8^ | 1.53 x 10^-8^ |
|  |  |  |  |  |  |  |
| ***GOF m*** | - |  |  |  |  |  |
| ***GOF f*** | F_(20;22)_ = 1.34  p = 0.4993 | - |  |  |  |  |
| ***+/+ m*** | F_(20; 20)_ = 5.21  p = 0.0005 | F_(22; 20)_ = 3.87  p =0.0034 | - |  |  |  |
| ***+/+ f*** | F_(20; 22)_ = 3.05  p = 0.0127 | F_(22; 22)_ = 2.27  p = 0.0600 | F_(20; 22)_ = 0.58  p = 0.2330 | - |  |  |
| ***LOF m*** | F_(20; 32)_ = 1.25  p = 0.5530 | F_(22; 32)_ = 0.93  p = 0.8820 | F_(20; 32)_ = 0.24  p = 0.0014 | F_(22;32)_ = 0.41  p = 0.032 | - |  |
| ***LOF f*** | F_(20; 26)_ = 1.20  p = 0.6480 | F_(22; 26)_ = 0.89  p = 0.7990 | F_(20; 26)_ = 0.23  p = 0.0013 | F_(22; 26)_ = 0.39  p = 0.029 | F_(32; 26)_ = 0.95  p = 0.9010 | - |
