# Supplementary material for: Developmental Stability: A Major Role for Cyclin G in Drosophila melanogaster
Source: PLoS Genet. 2011 Oct 6;7(10):e1002314. doi: 10.1371/journal.pgen.1002314 (PMC3188557; doi:10.1371/journal.pgen.1002314)
Supplement: Table S8 — Effects on cell size and cell number FA. FA values for each genotype and sex is provided, as well as the statistical tests of comparison with the controls (F tests). The correlation between cell size and cell number FA with wing size FA were computed. Both Pearson parametric correlation coefficient and Spearman non parametric correlation were computed. Only Pearson's r values are shown with the corresponding test of statistical significance, as both tests provided very similar results. Df = degrees of freedom; MS = mean squares; F = Fisher's F value; * = p<0.05; ** = p<0.01; *** = p<0.001; ns = non significant. (DOC) [file pgen.1002314.s012.doc]

**Table S8: Effects on cell size and cell number FA.**

| **Cell size** | |  |  |  |  |  |
| --- | --- | --- | --- | --- | --- | --- |
|  |  |  |  |  |  |  |
| **Genotype** | ***+/+ f*** | ***+/+ m*** | ***GOF f*** | ***GOF m*** | ***LOF f*** | ***LOF m*** |
| **FA (FA4)** | 9.39 x 10^-9^ | 1.42 x 10^-8^ | 5.74 x 10^-8^ | 3.80 x 10^-8^ | 5.55 x 10^-8^ | 3.53 x 10^-8^ |
|  |  |  | 6.11 | 2.71 | 5.91 | 2.49 |
|  |  |  |  |  |  |  |
| **F test** | **comparison** | **F** | **df num** | **df den** | **P-value** | **p-adj** |
|  | *GOF m - +/+ m* | 2.67 | 20 | 20 | 0.033 | 0.0663 (ns) |
|  | *GOF f - +/+ f* | 6.11 | 22 | 22 | 7.58 x 10^-5^ | 0.0003 (***) |
|  | *LOF m - +/+m* | 2.49 | 32 | 20 | 0.036 | 0.0663 (ns) |
|  | *LOF f - +/+f* | 5.91 | 26 | 22 | 7.50 x 10^-5^ | 0.0003 (***) |
|  |  |  |  |  |  |  |
| **Cell number** | |  |  |  |  |  |
|  |  |  |  |  |  |  |
| **Genotype** | ***+/+ f*** | ***+/+ m*** | ***GOF f*** | ***GOF m*** | ***LOF f*** | ***LOF m*** |
| **FA (FA4)** | 868382.9 | 473199.9 | 1629954.1 | 1237963.9 | 369746.1 | 272271.8 |
|  |  |  | 1.87 | 2.60 | 0.42 | 0.57 |
|  |  |  |  |  |  |  |
| **F test** | **comparison** | **F** | **df num** | **df den** | **P-value** | **p-adj** |
|  | *GOF m - +/+ m* | 2.62 | 20 | 20 | 0.037 | 0.1480 (ns) |
|  | *GOF f - +/+ f* | 1.88 | 22 | 22 | 0.148 | 0.2950 (ns) |
|  | *LOF m - +/+m* | 0.58 | 32 | 20 | 0.159 | 0.2950 (ns) |
|  | *LOF f - +/+f* | 0.43 | 26 | 22 | 0.039 | 0.1480 (ns) |
|  |  |  |  |  |  |  |
| **Correlations** | |  |  |  |  |  |
|  |  |  |  |  |  |  |
| **CS asym. *vs* WS asym.** | | **Genotype** | **Pearson's r** | **t** | **df** | **P-value** |
|  |  | *GOF m* | 0.65 | 3.75 | 19 | 0.001 (**) |
|  |  | *GOF f* | 0.54 | 2.91 | 21 | 0.008 (**) |
|  |  | *+/+ m* | -0.13 | -0.57 | 19 | 0.573 (ns) |
|  |  | *+/+ f* | 0.14 | 0.65 | 21 | 0.523 (ns) |
|  |  |  |  |  |  |  |
| **N asym. *vs* WS asym.** | | **Genotype** | **Pearson's r** | **t** | **df** | **P-value** |
|  |  | *GOF m* | 0.11 | 0.48 | 19 | 0.637 (ns) |
|  |  | *GOF f* | 0.27 | 1.28 | 21 | 0.214 (ns) |
|  |  | *+/+ m* | -0.69 | -4.16 | 19 | 0.001 (**) |
|  |  | *+/+ f* | 0.2 | 0.93 | 21 | 0.361 (ns) |
